# Supplementary figures and images for: Discovery of a new class of reversible TEA domain transcription factor inhibitors with a novel binding mode
Source: eLife. 2022 Nov 18;11:e80210. doi: 10.7554/eLife.80210 (PMC9728997; doi:10.7554/eLife.80210)

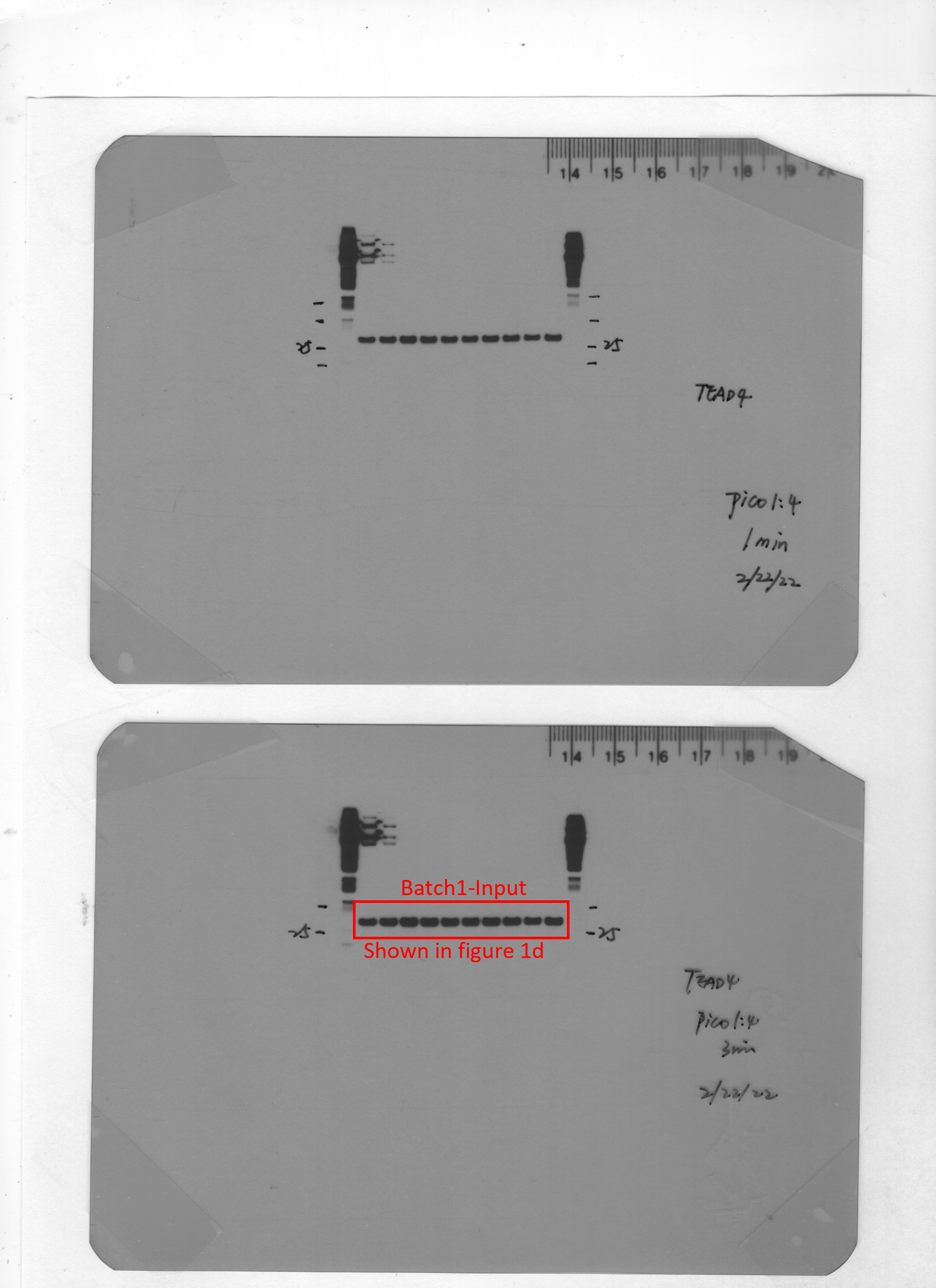

Supplement: Figure 1—source data 1. [file elife-80210-fig1-data1.zip › Figure 1D/TEAD4-IC50-Input-1 - labeled.tif]

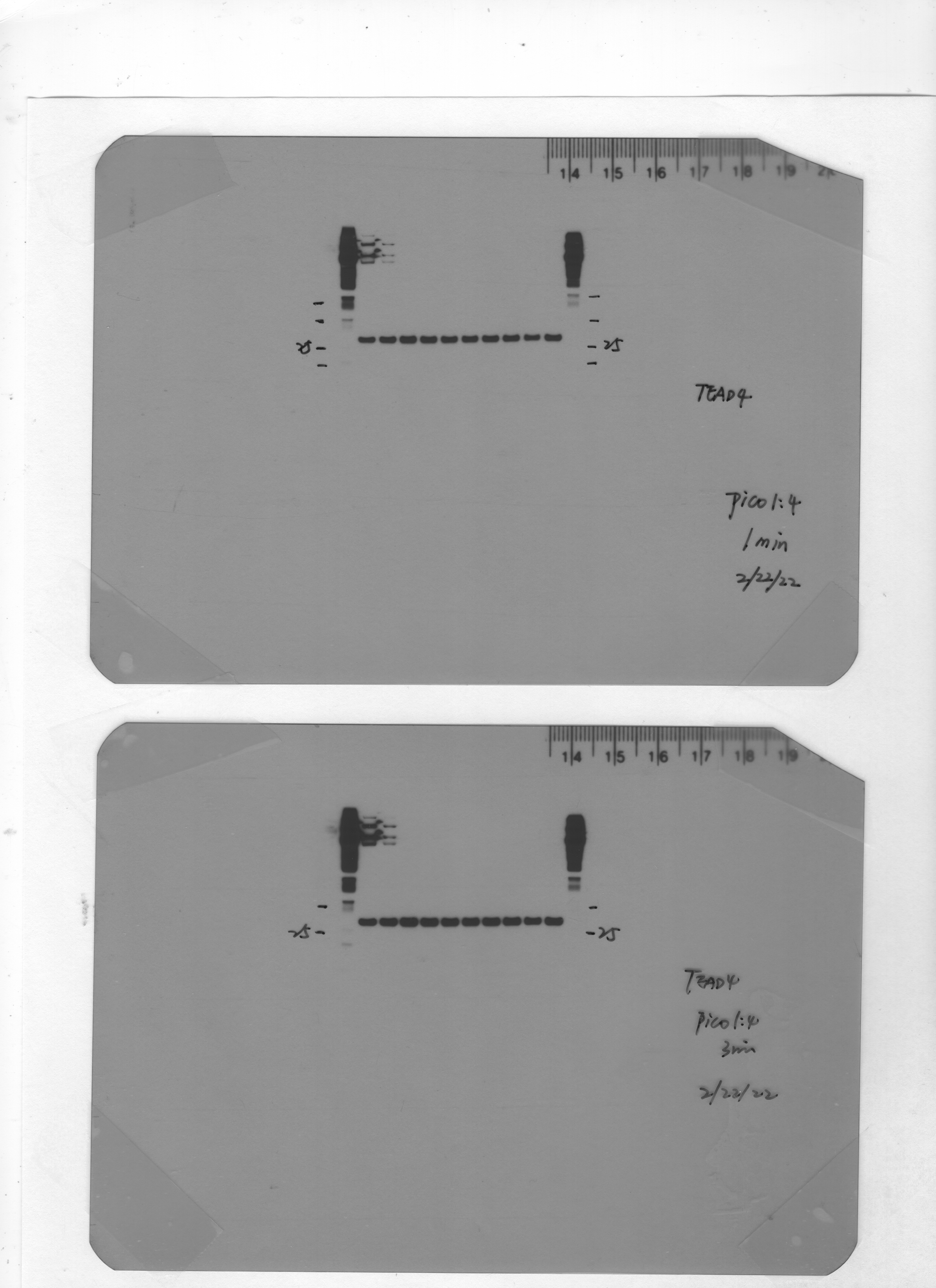

Supplement: Figure 1—source data 1. [file elife-80210-fig1-data1.zip › Figure 1D/TEAD4-IC50-Input-1.tif]

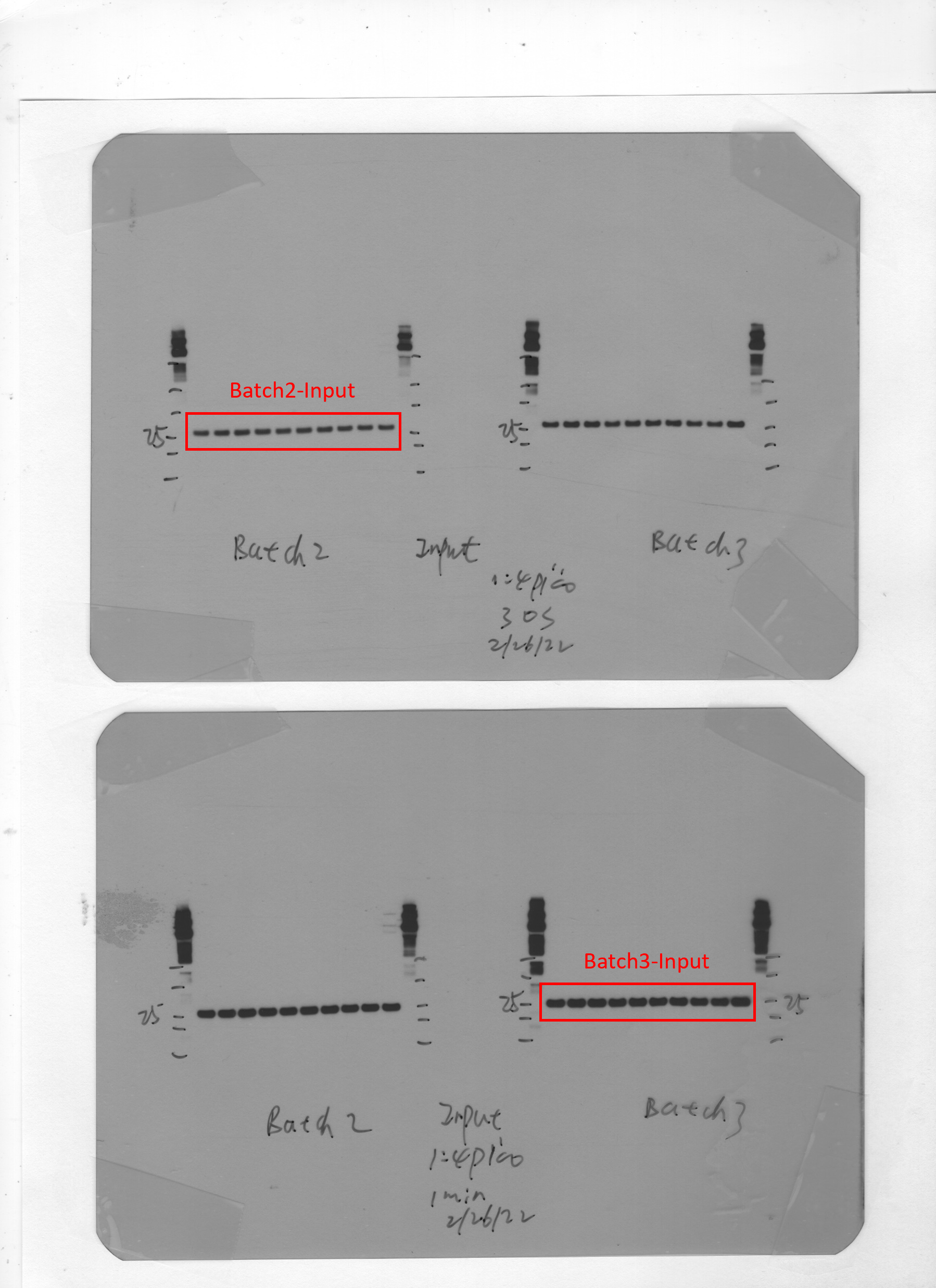

Supplement: Figure 1—source data 1. [file elife-80210-fig1-data1.zip › Figure 1D/TEAD4-IC50-Input-2 - labeled.tif]

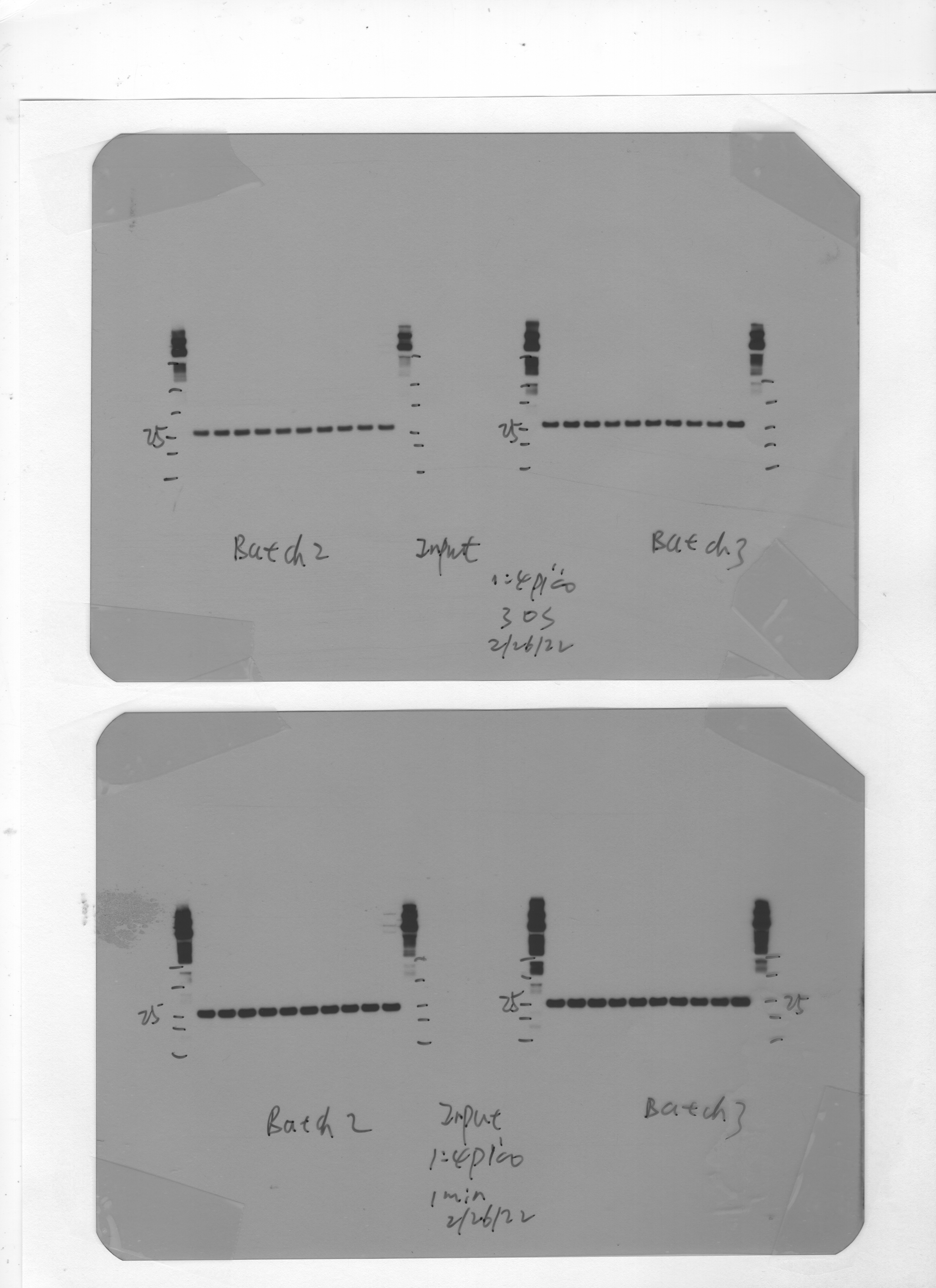

Supplement: Figure 1—source data 1. [file elife-80210-fig1-data1.zip › Figure 1D/TEAD4-IC50-Input-2.tif]

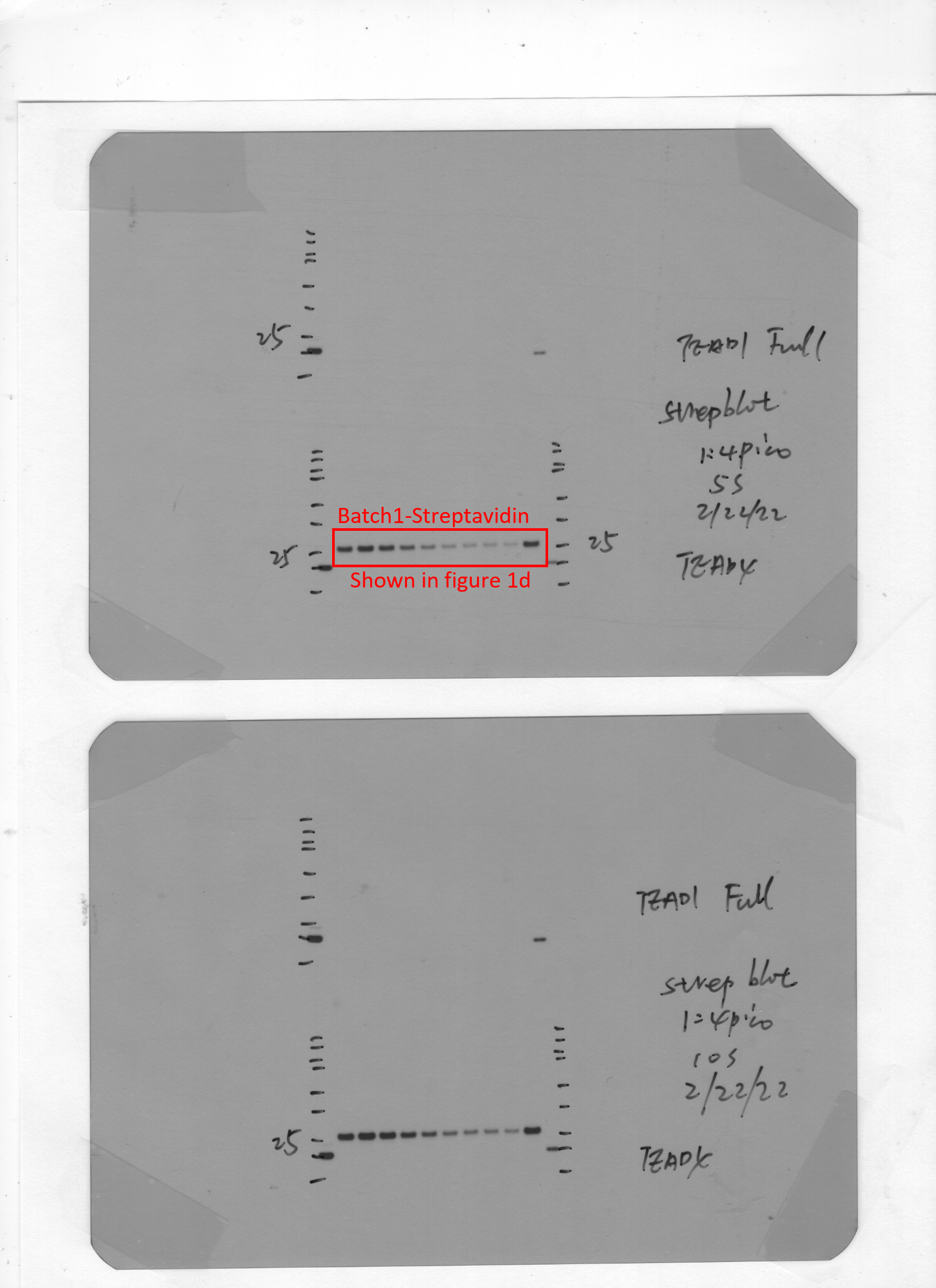

Supplement: Figure 1—source data 1. [file elife-80210-fig1-data1.zip › Figure 1D/TEAD4-IC50-strep-1 - labeled.tif]

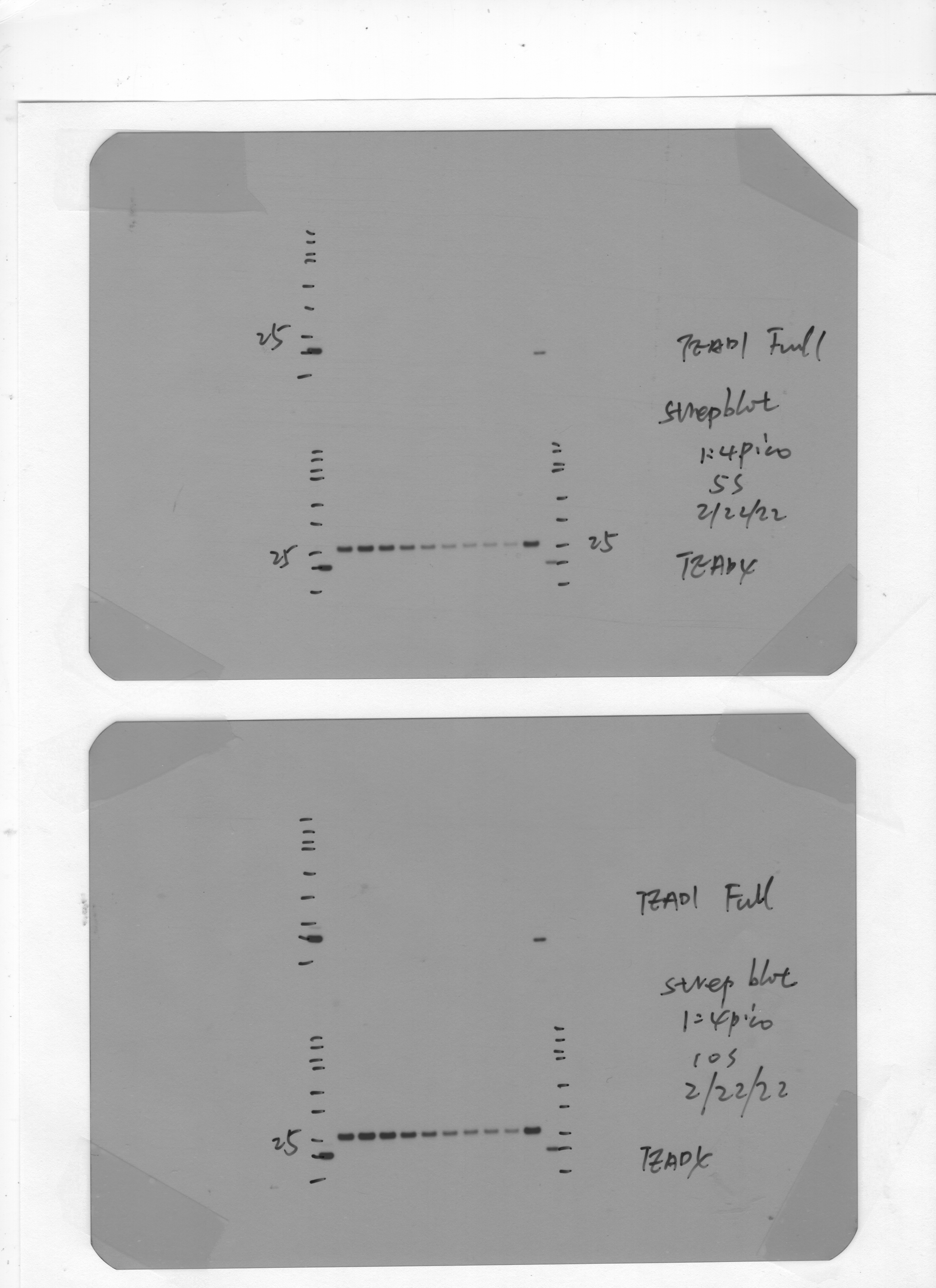

Supplement: Figure 1—source data 1. [file elife-80210-fig1-data1.zip › Figure 1D/TEAD4-IC50-strep-1.tif]

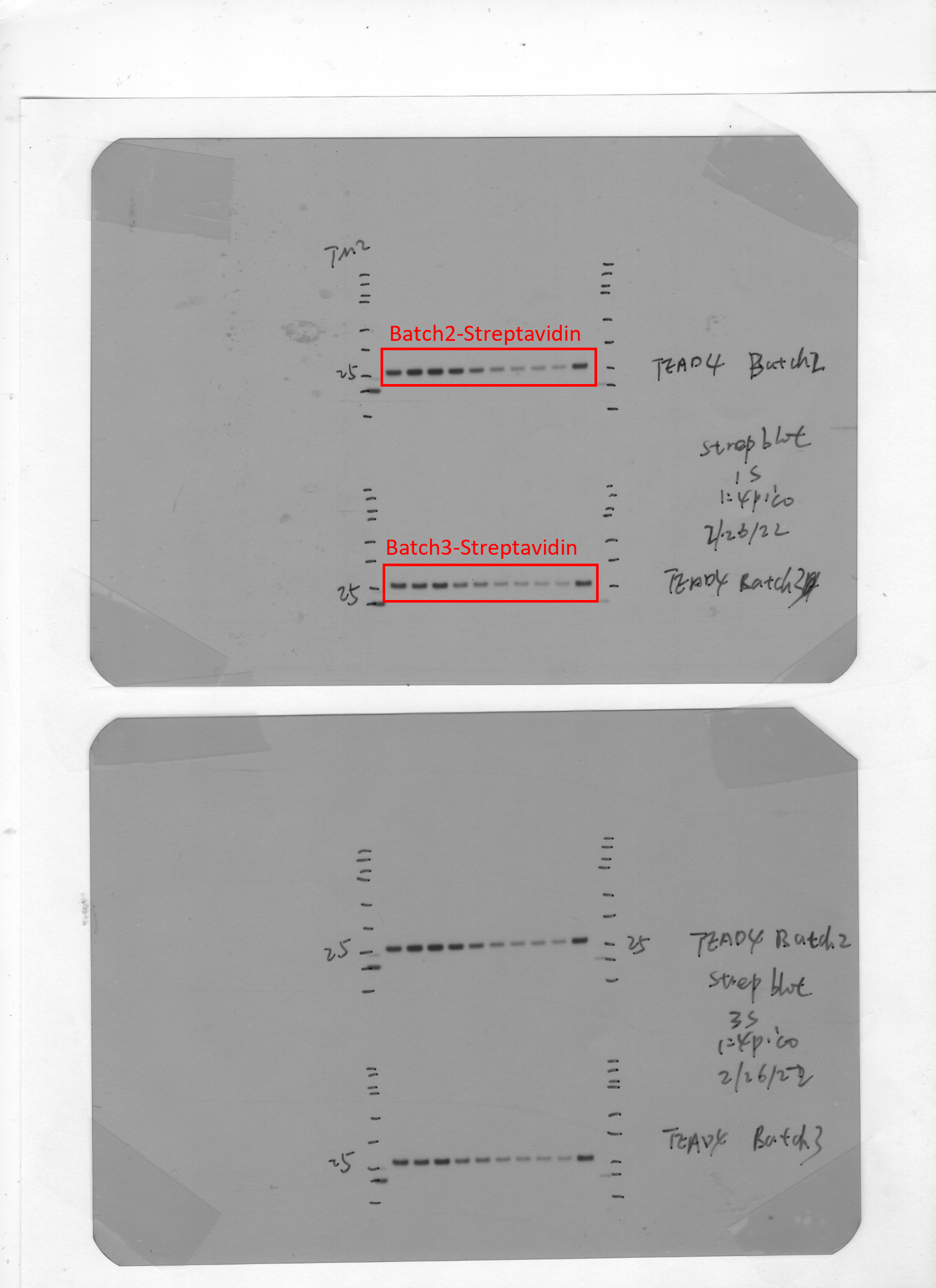

Supplement: Figure 1—source data 1. [file elife-80210-fig1-data1.zip › Figure 1D/TEAD4-IC50-strep-2 - labeled.tif]

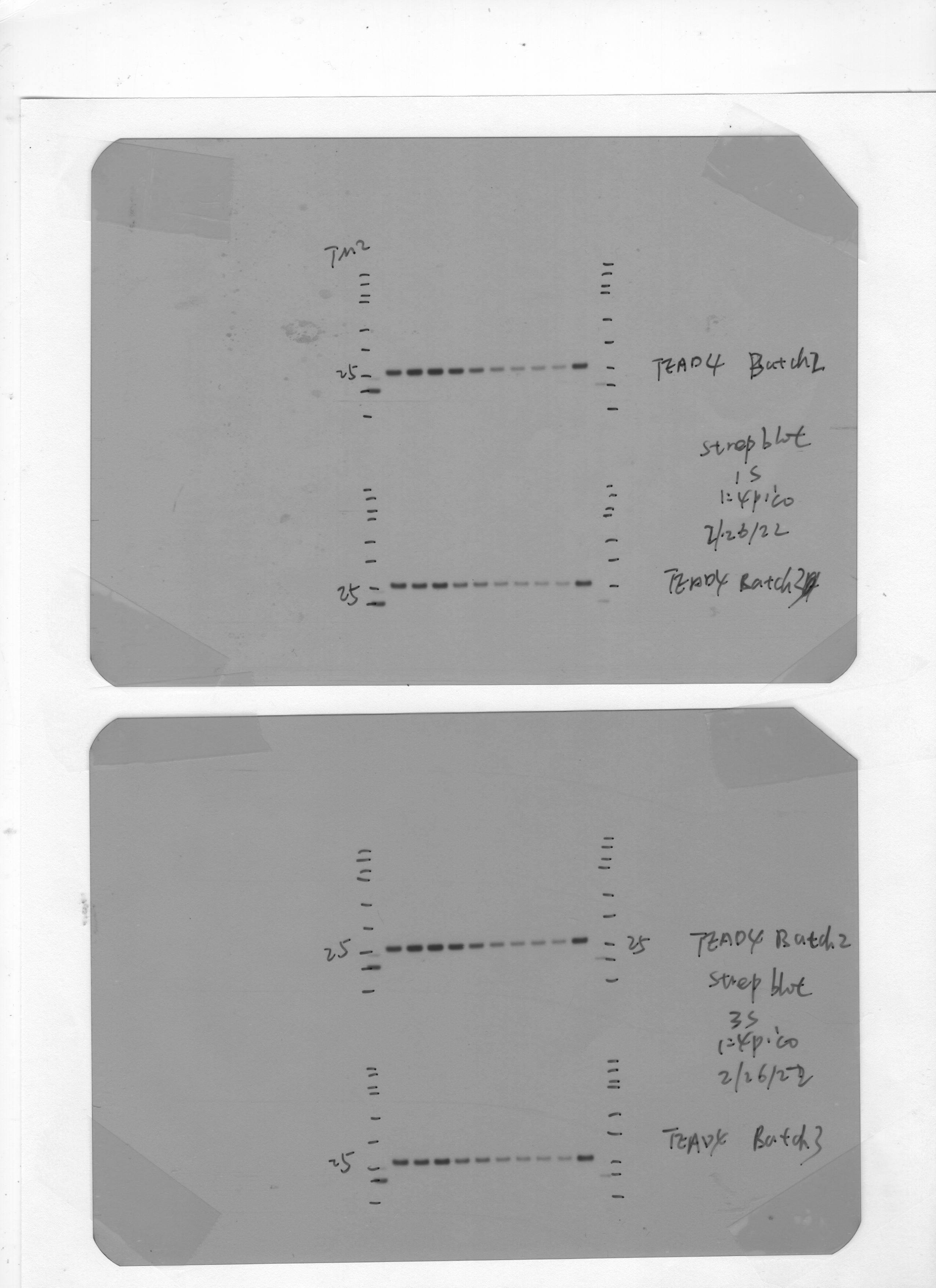

Supplement: Figure 1—source data 1. [file elife-80210-fig1-data1.zip › Figure 1D/TEAD4-IC50-strep-2.tif]

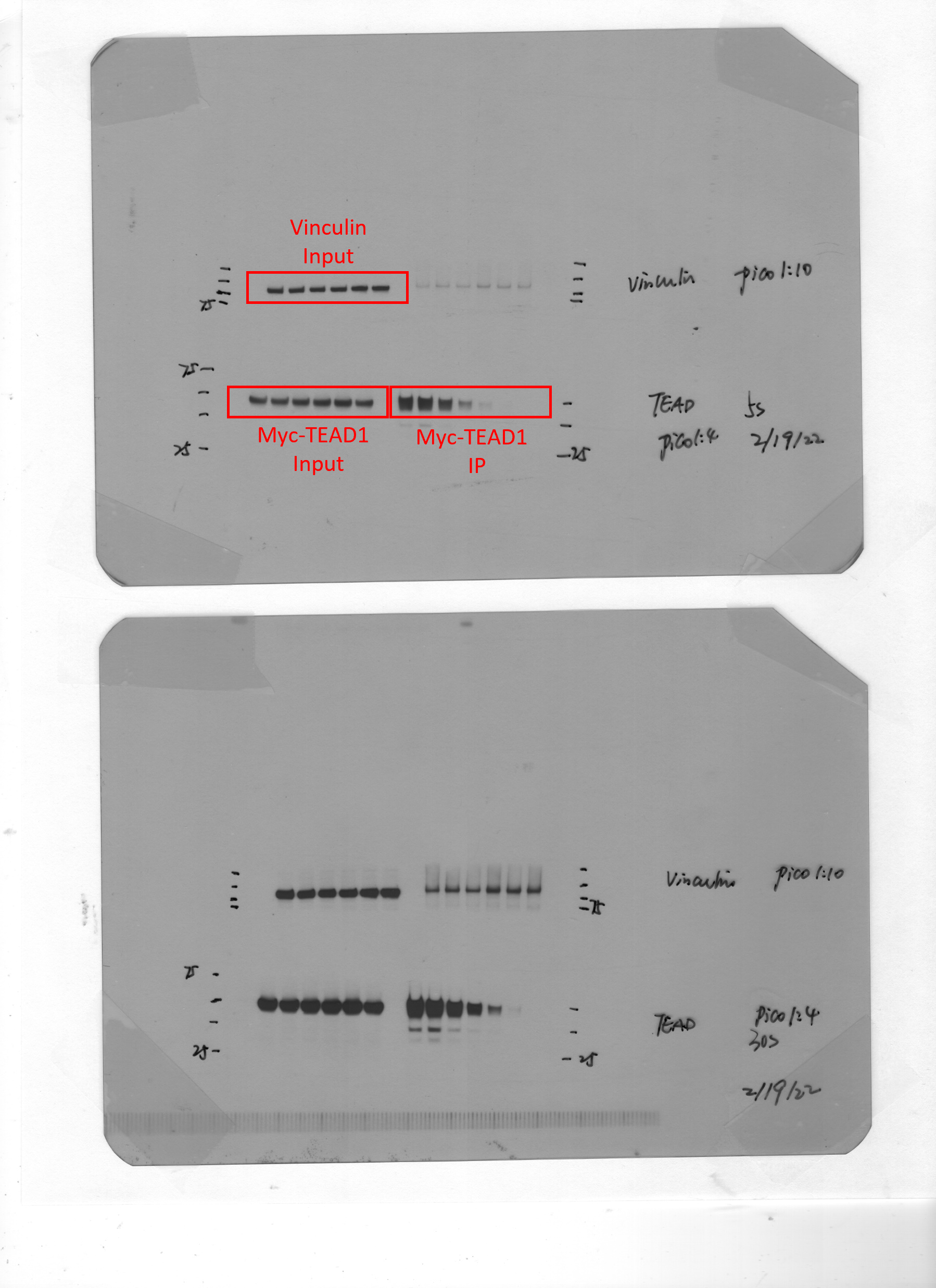

Supplement: Figure 1—source data 1. [file elife-80210-fig1-data1.zip › Figure 1E/Figure 1E-labeled.tif]

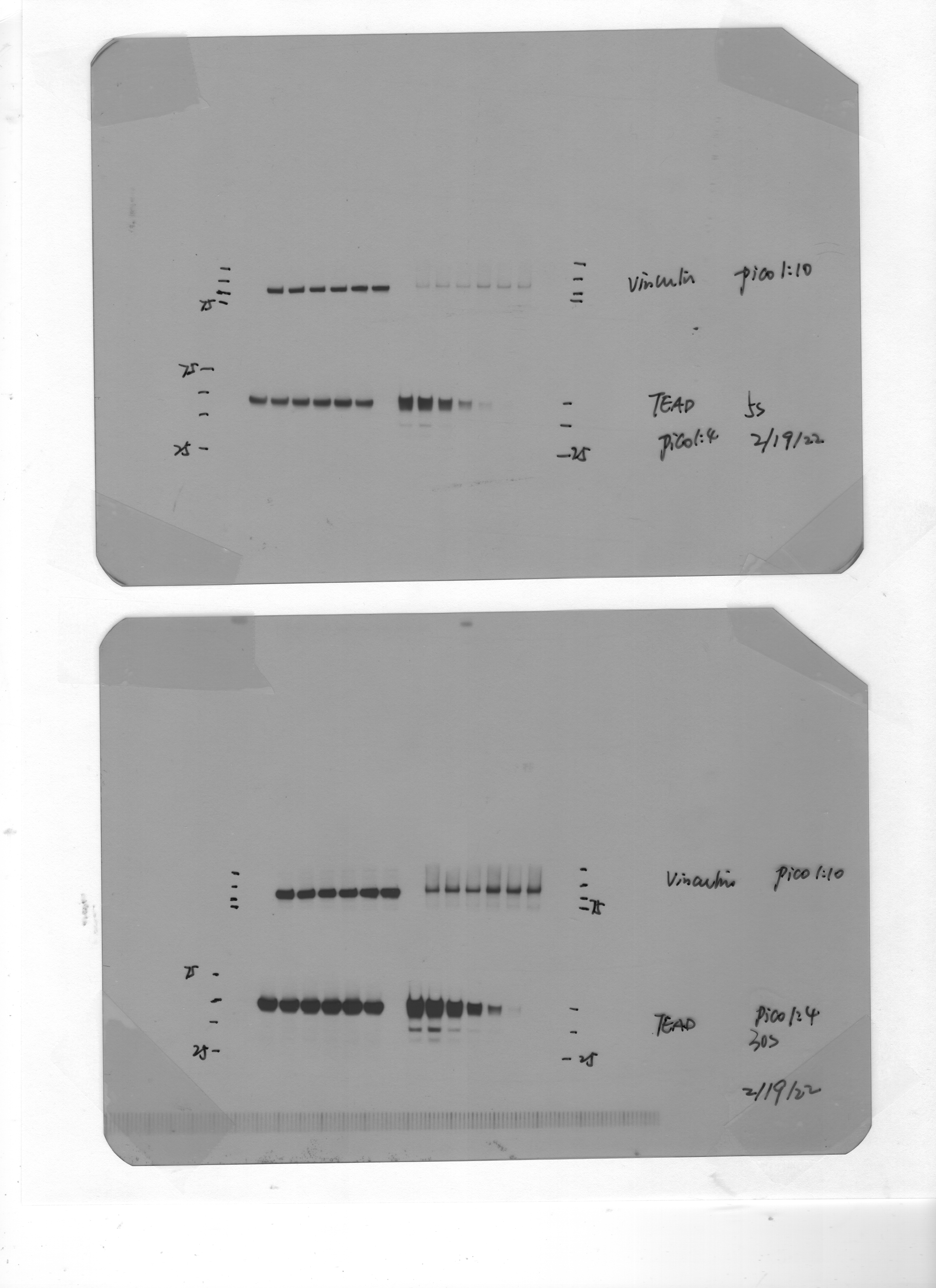

Supplement: Figure 1—source data 1. [file elife-80210-fig1-data1.zip › Figure 1E/Figure 1E.tif]

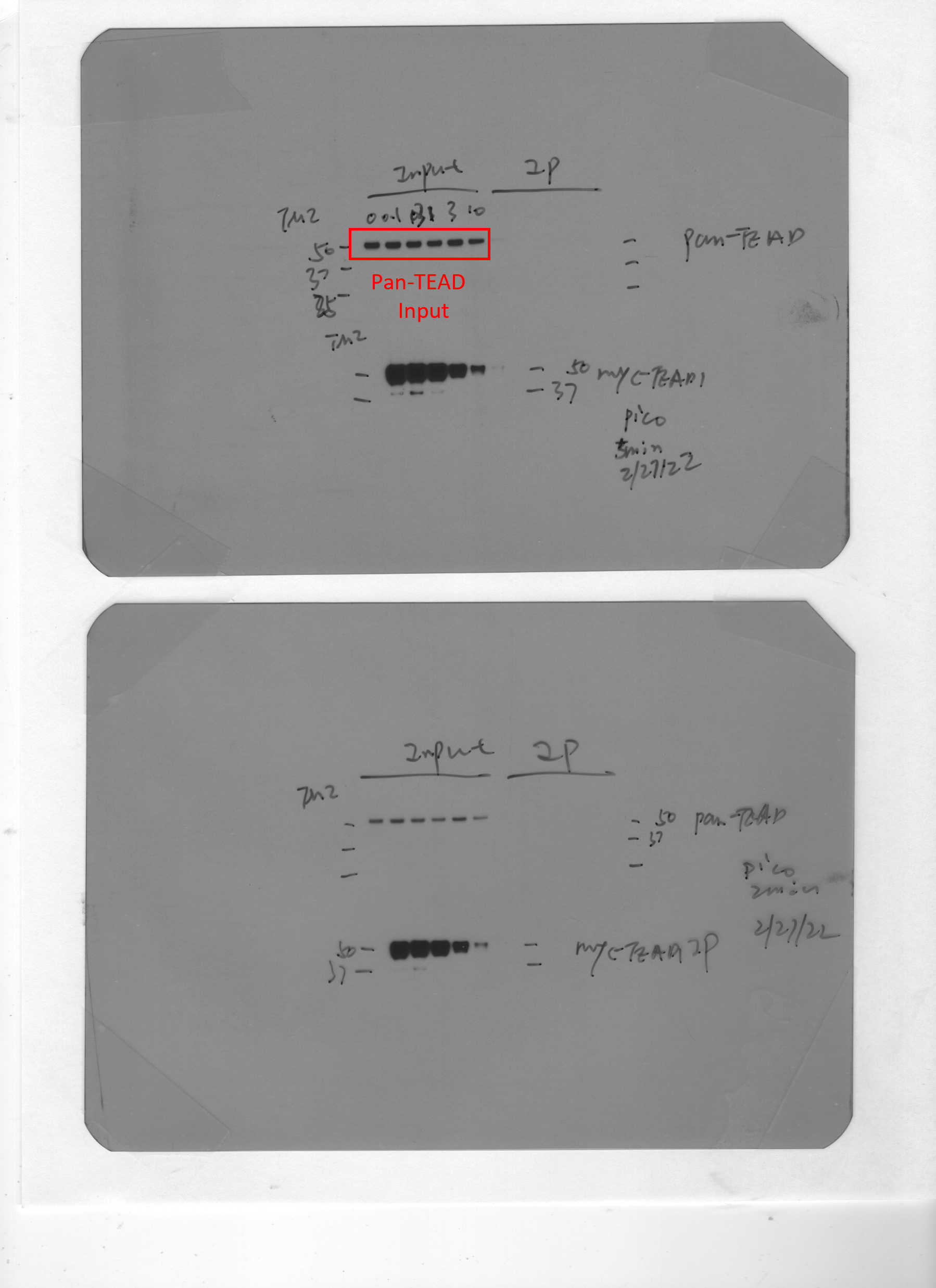

Supplement: Figure 1—source data 1. [file elife-80210-fig1-data1.zip › Figure 1F/Figure 1F-Input-panTEAD-labled.tif]

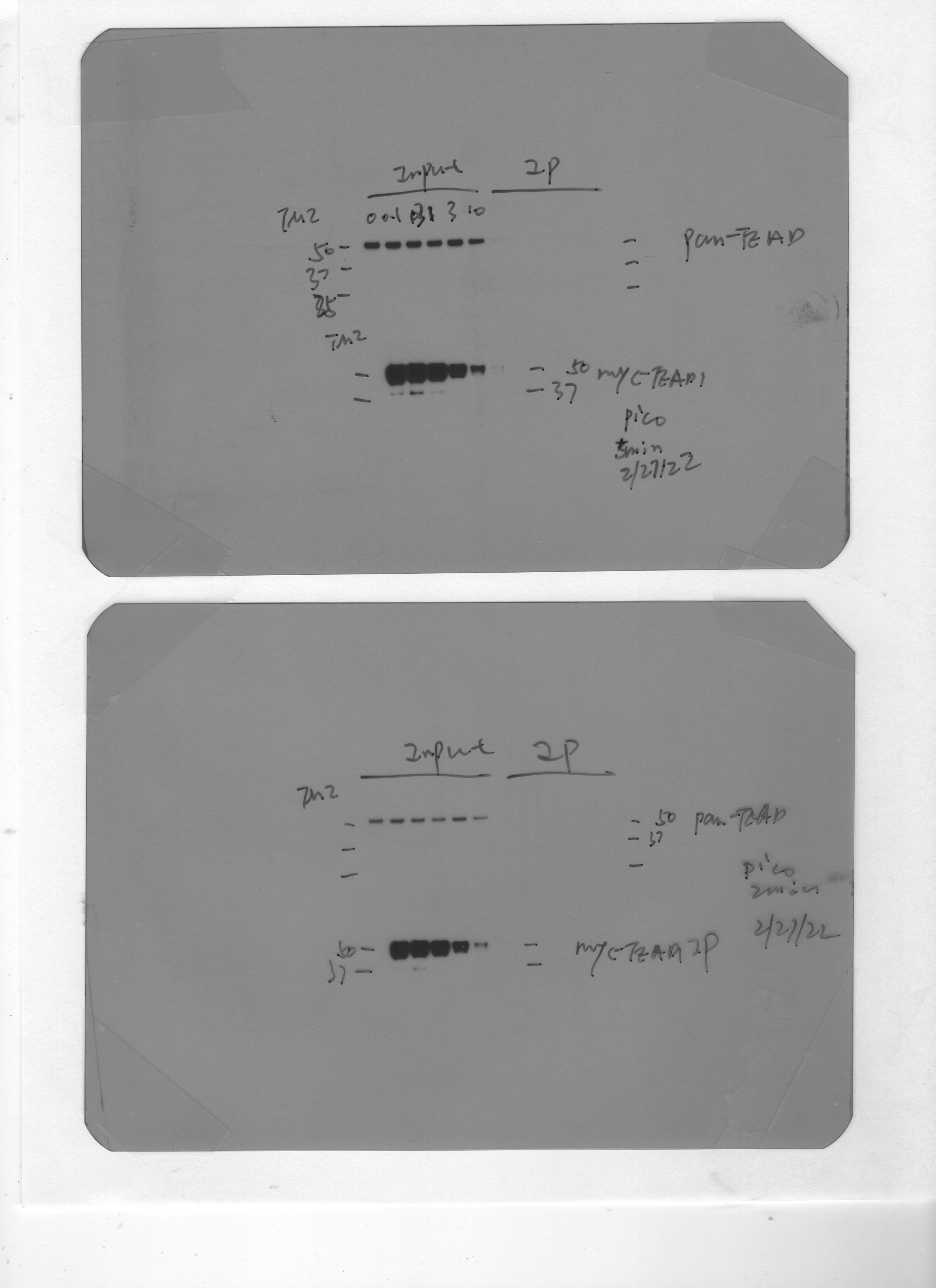

Supplement: Figure 1—source data 1. [file elife-80210-fig1-data1.zip › Figure 1F/Figure 1F-Input-panTEAD.tif]

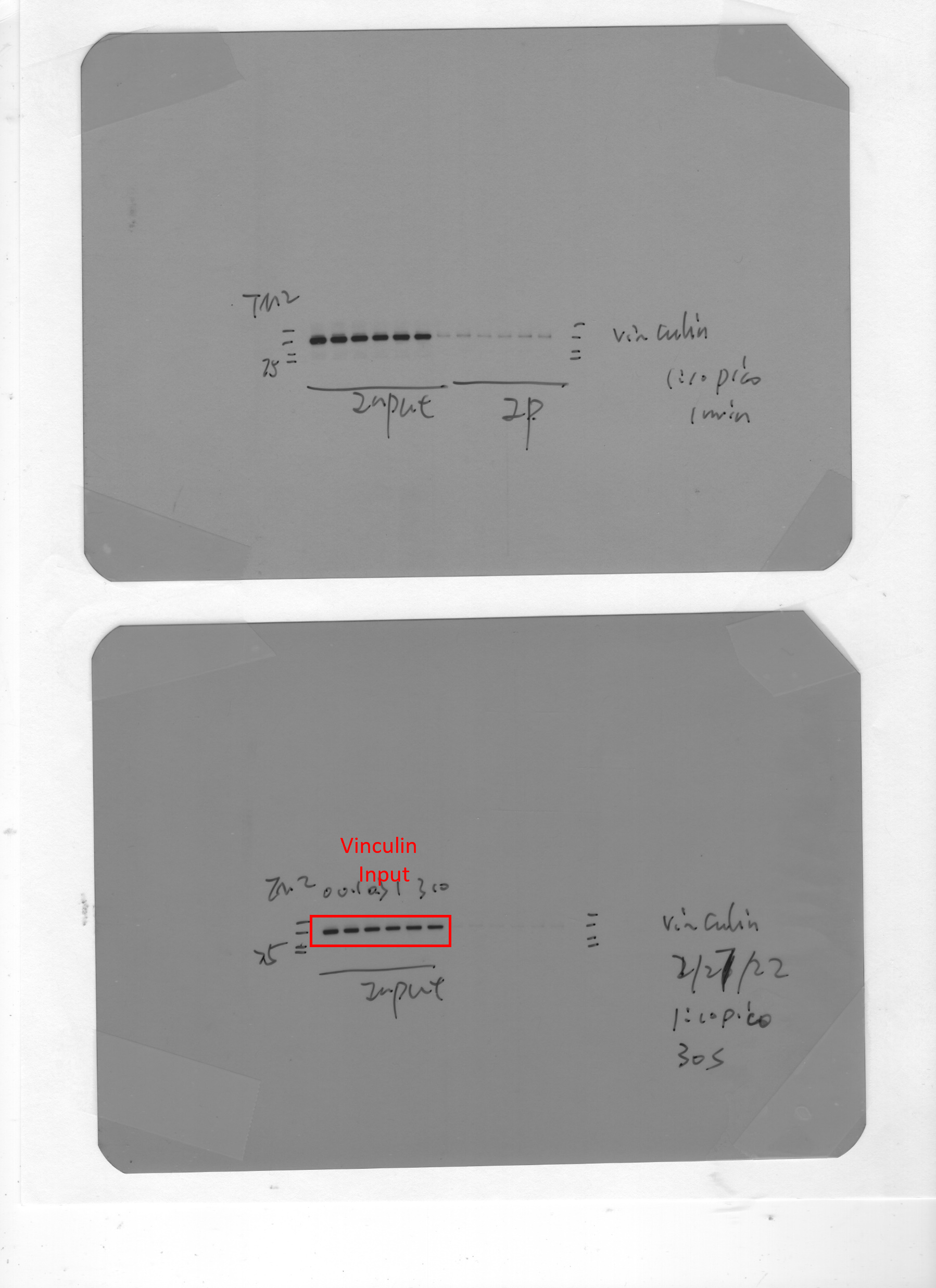

Supplement: Figure 1—source data 1. [file elife-80210-fig1-data1.zip › Figure 1F/Figure 1F-Input-Vinculin-labled.tif]

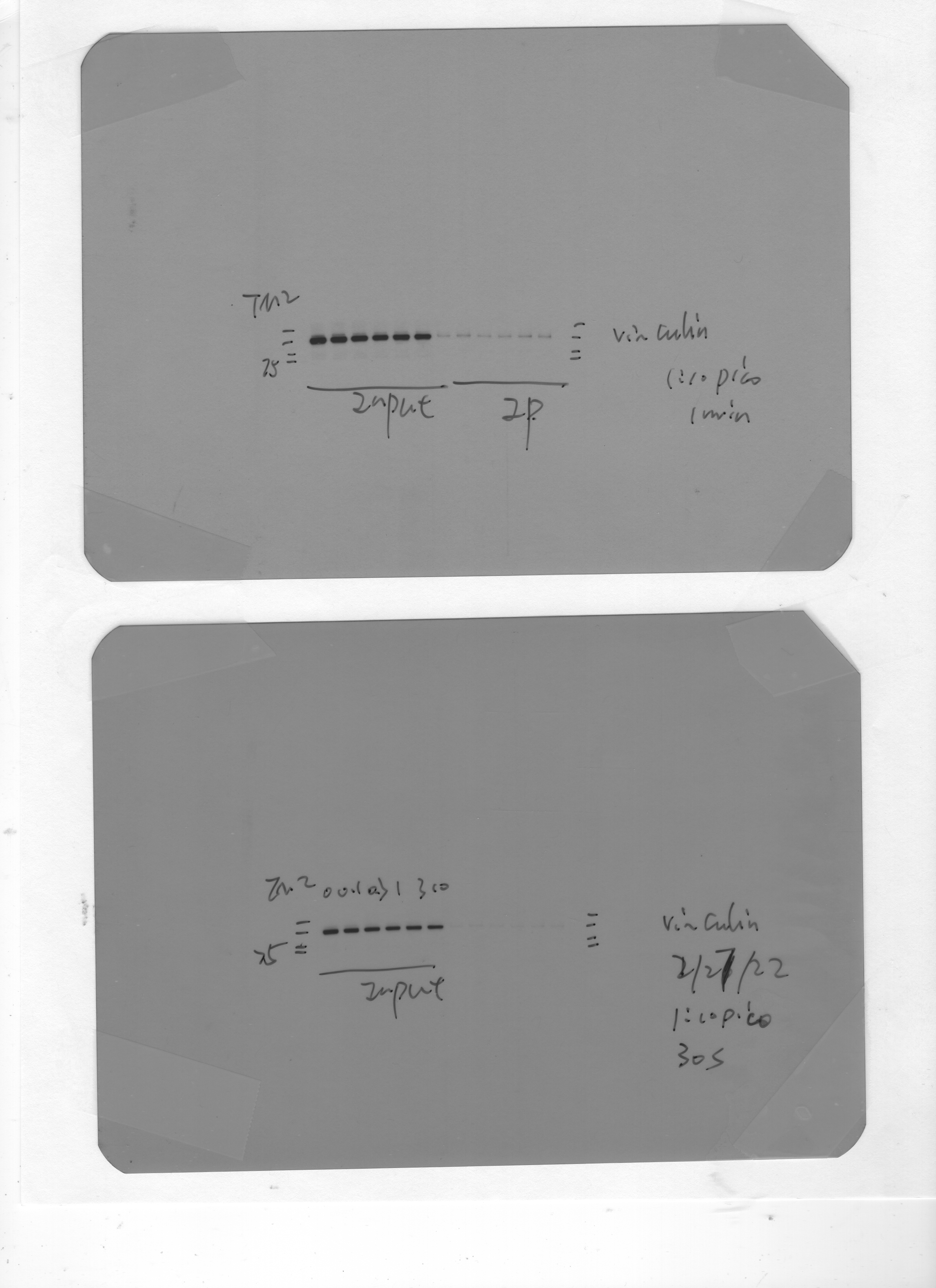

Supplement: Figure 1—source data 1. [file elife-80210-fig1-data1.zip › Figure 1F/Figure 1F-Input-Vinculin.tif]

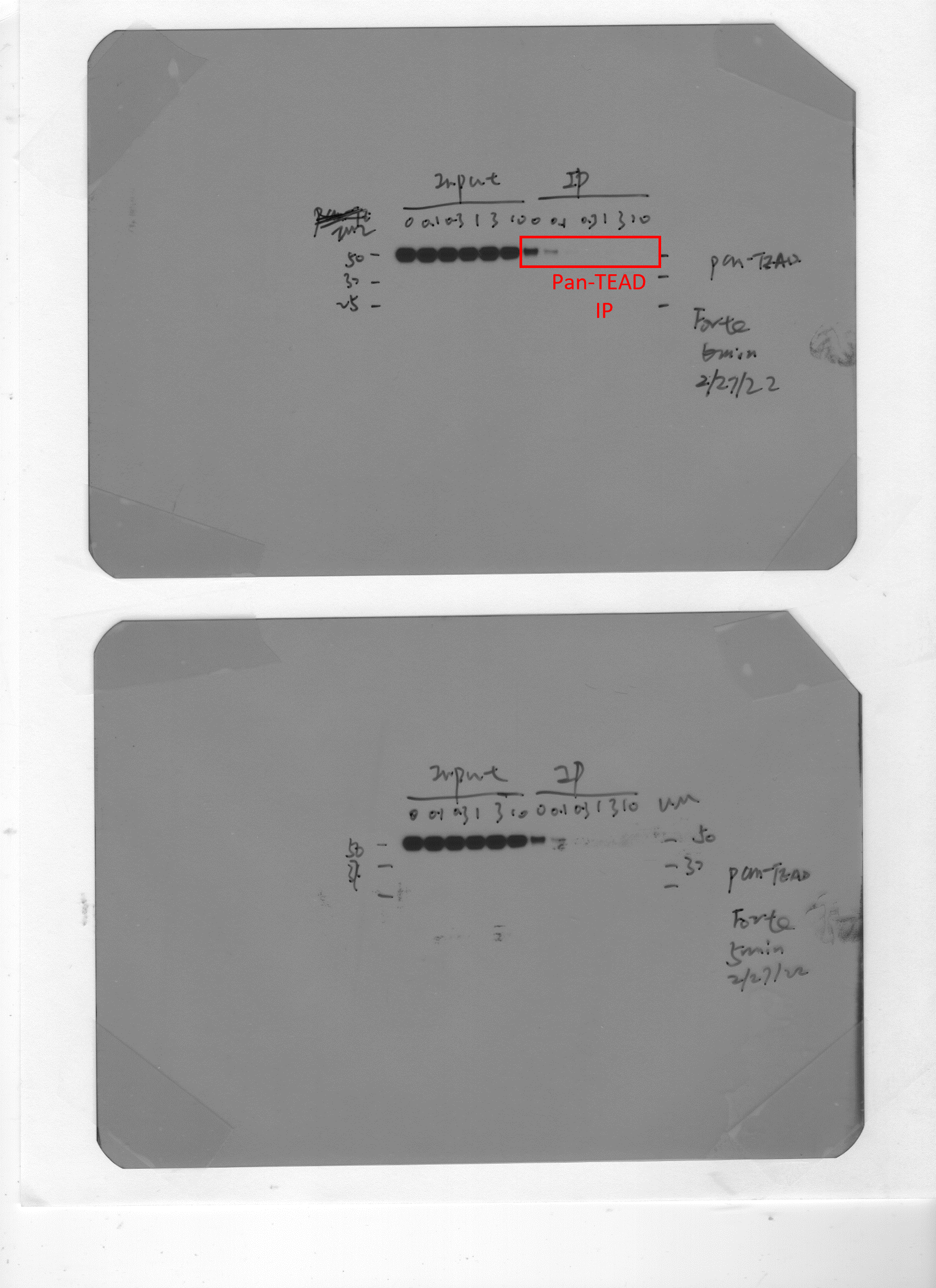

Supplement: Figure 1—source data 1. [file elife-80210-fig1-data1.zip › Figure 1F/Figure 1F-IP-pan-TEAD-labled.tif]

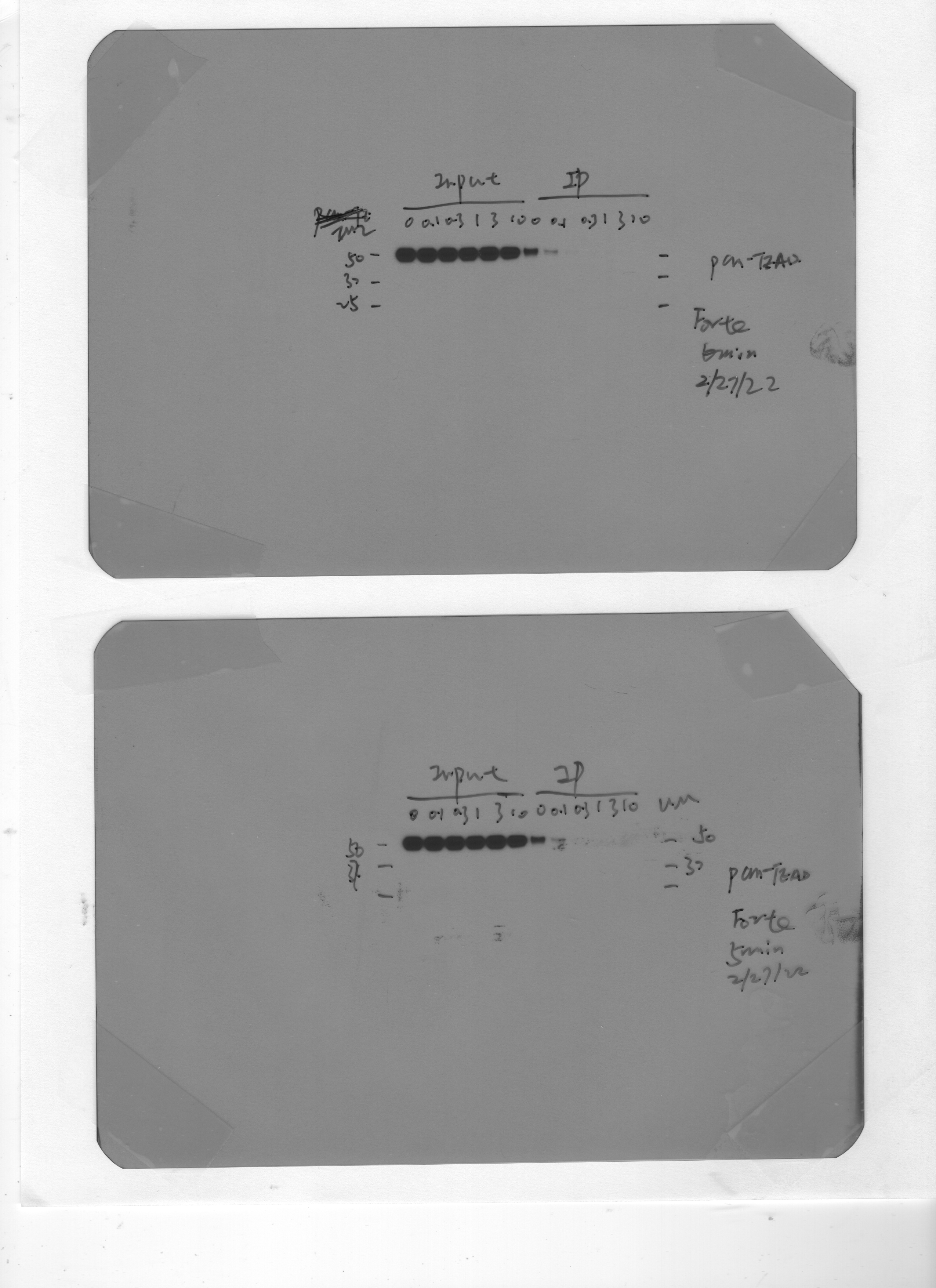

Supplement: Figure 1—source data 1. [file elife-80210-fig1-data1.zip › Figure 1F/Figure 1F-IP-panTEAD.tif]

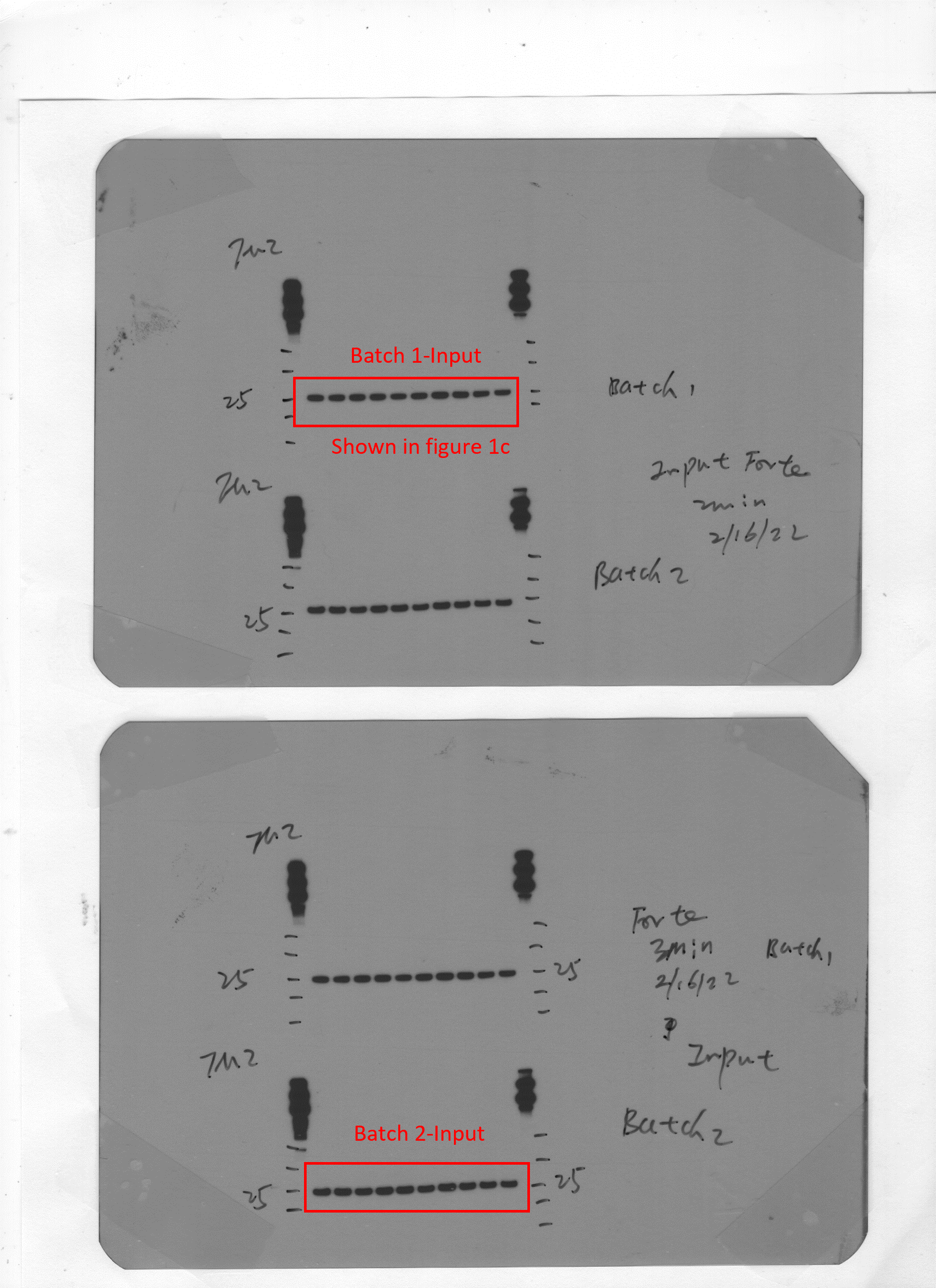

Supplement: Figure 1—source data 1. [file elife-80210-fig1-data1.zip › Figure 1C/TEAD2-IC50-Input-1 - labeled.tif]

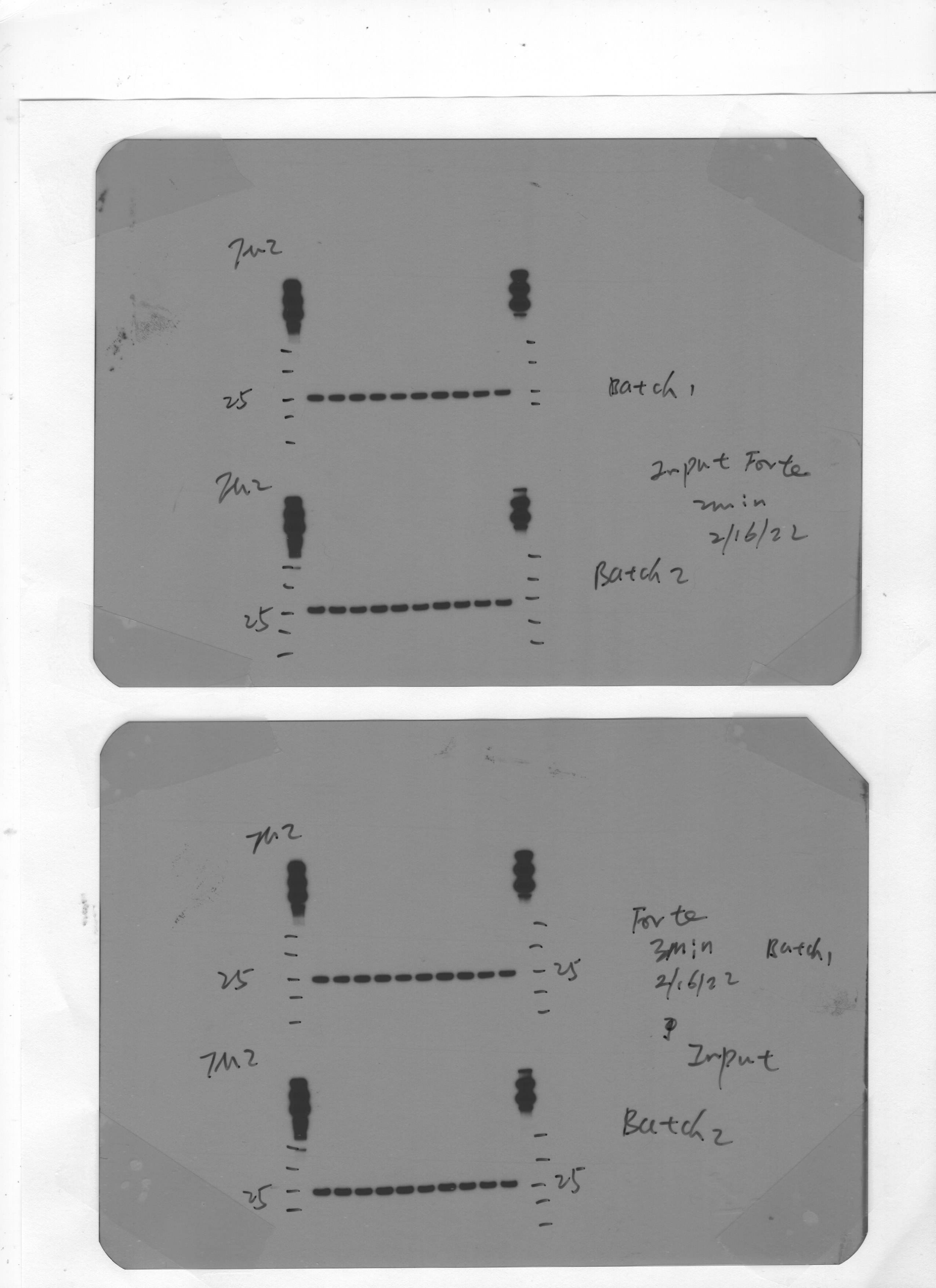

Supplement: Figure 1—source data 1. [file elife-80210-fig1-data1.zip › Figure 1C/TEAD2-IC50-Input-1.tif]

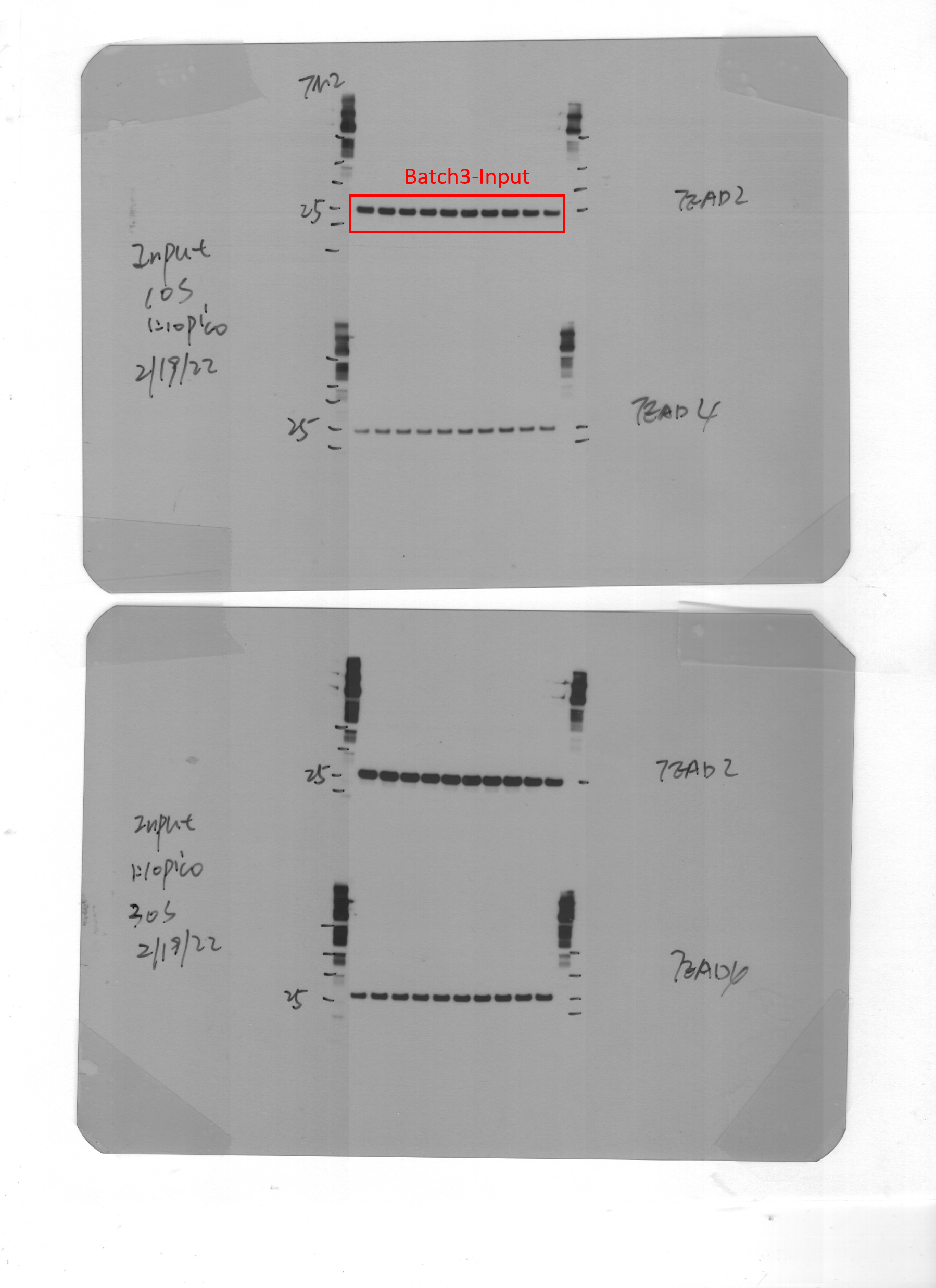

Supplement: Figure 1—source data 1. [file elife-80210-fig1-data1.zip › Figure 1C/TEAD2-IC50-Input-2 - labled.tif]

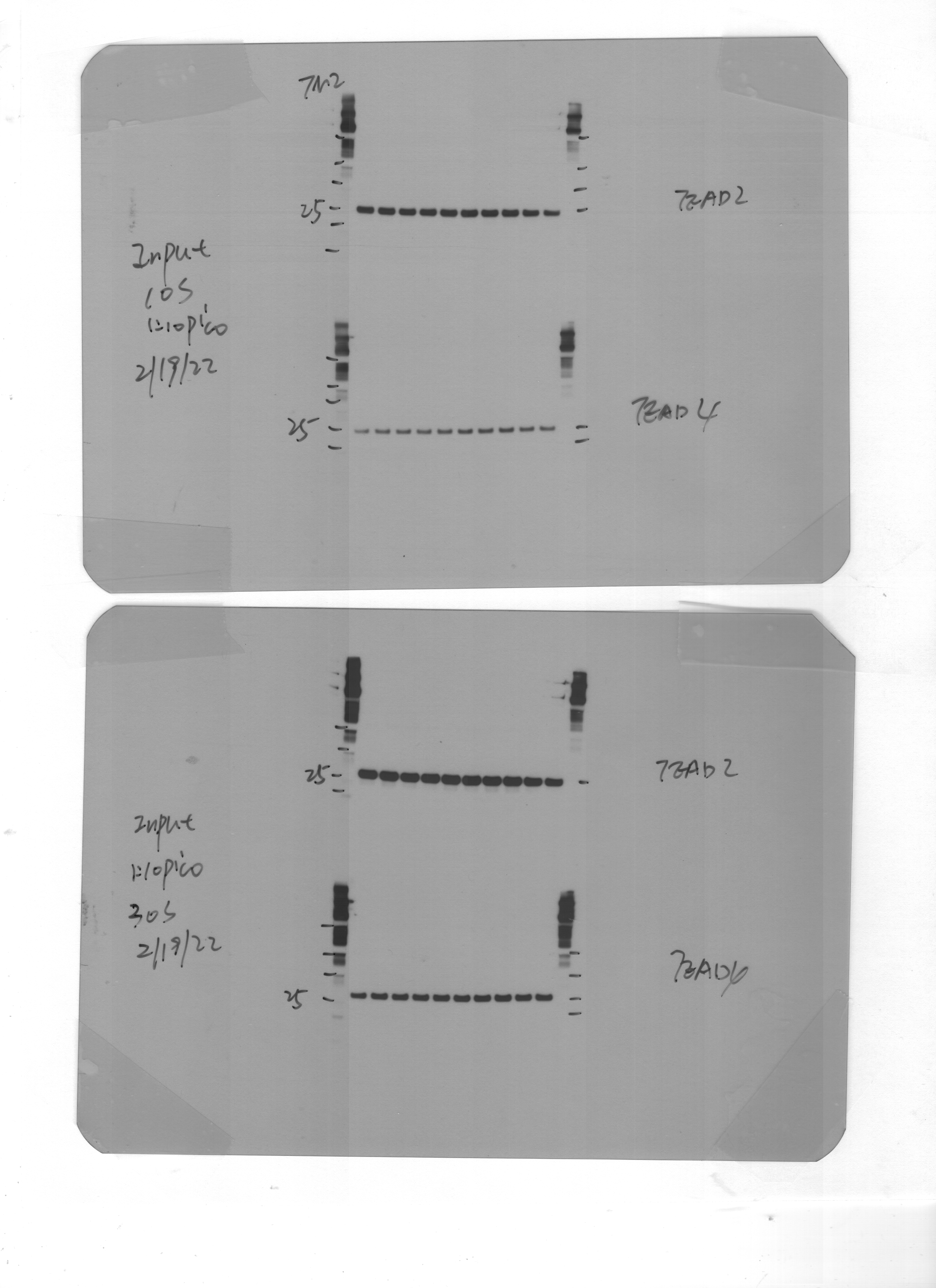

Supplement: Figure 1—source data 1. [file elife-80210-fig1-data1.zip › Figure 1C/TEAD2-IC50-Input-2.tif]

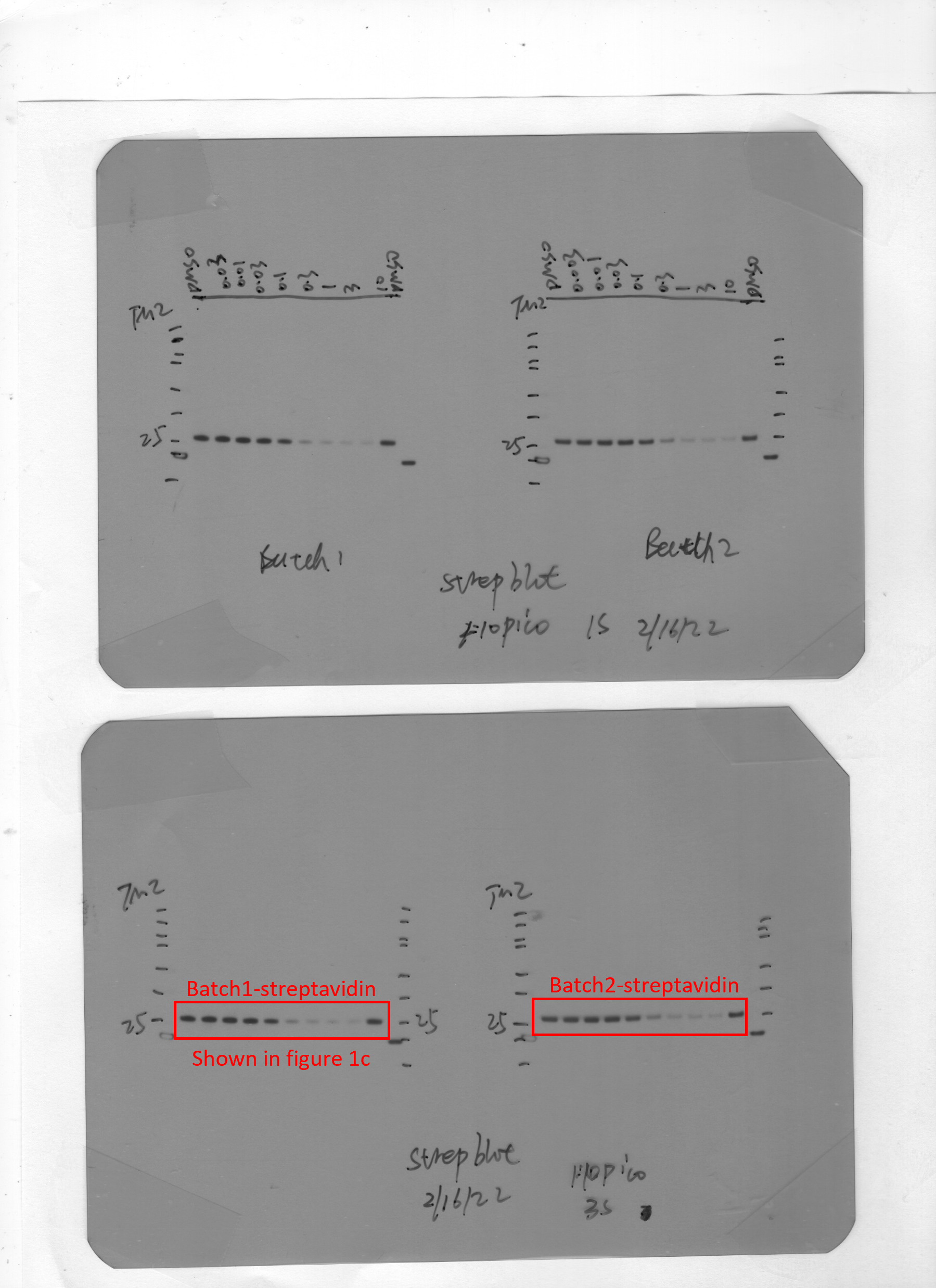

Supplement: Figure 1—source data 1. [file elife-80210-fig1-data1.zip › Figure 1C/TEAD2-IC50-strep-1 -labled.tif]

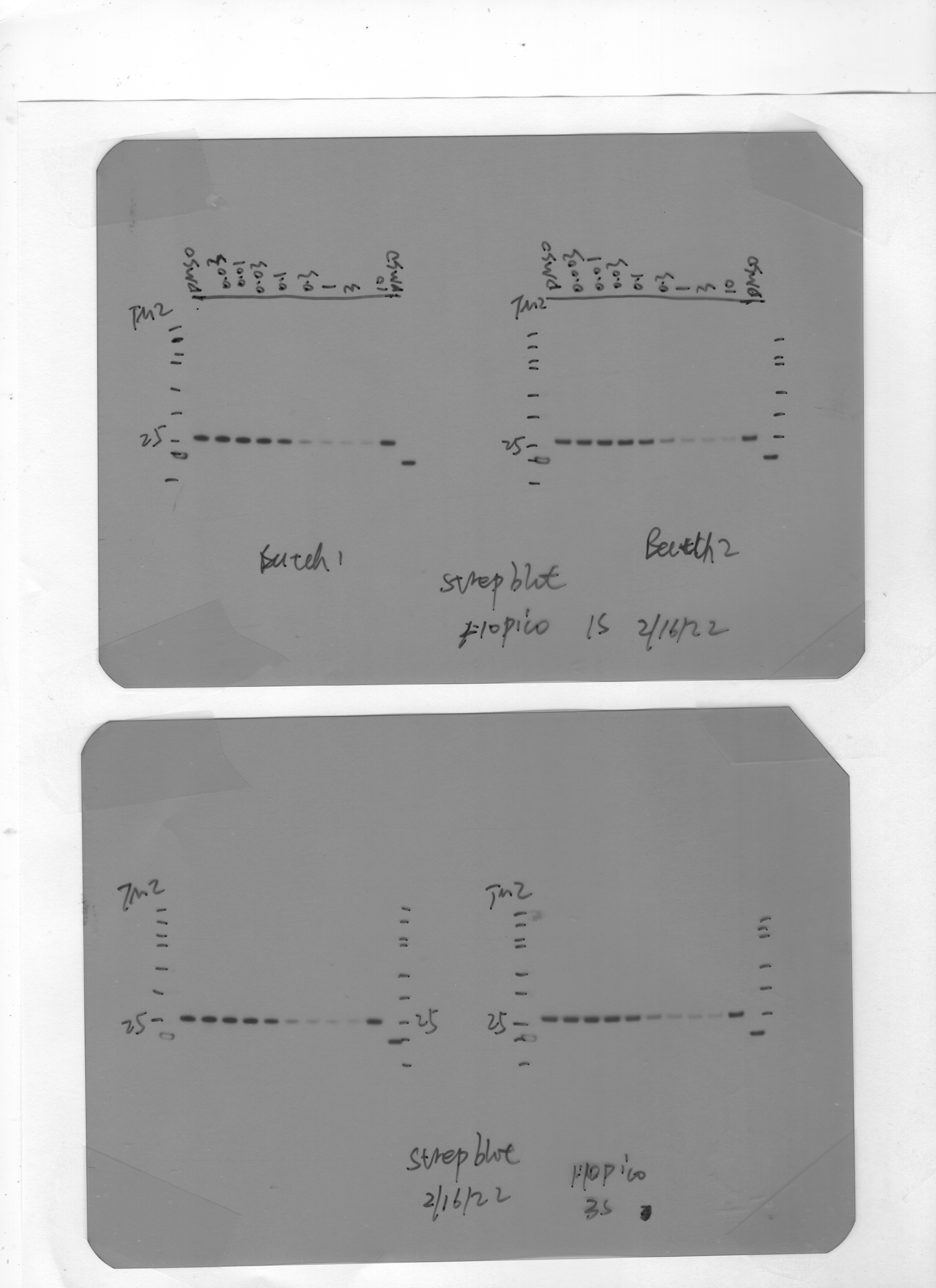

Supplement: Figure 1—source data 1. [file elife-80210-fig1-data1.zip › Figure 1C/TEAD2-IC50-strep-1.tif]

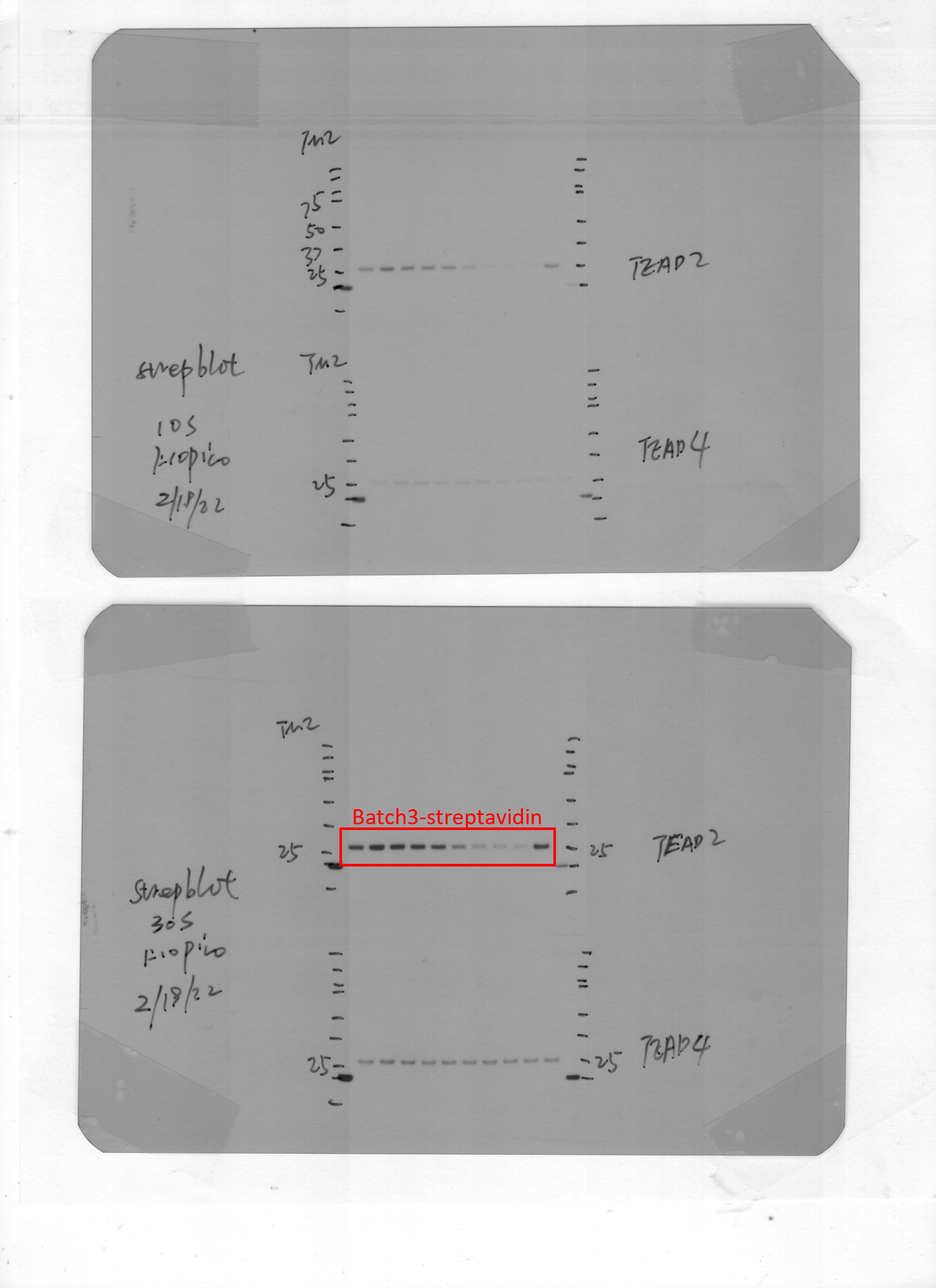

Supplement: Figure 1—source data 1. [file elife-80210-fig1-data1.zip › Figure 1C/TEAD2-IC50-strep-2 -labeled.tif]

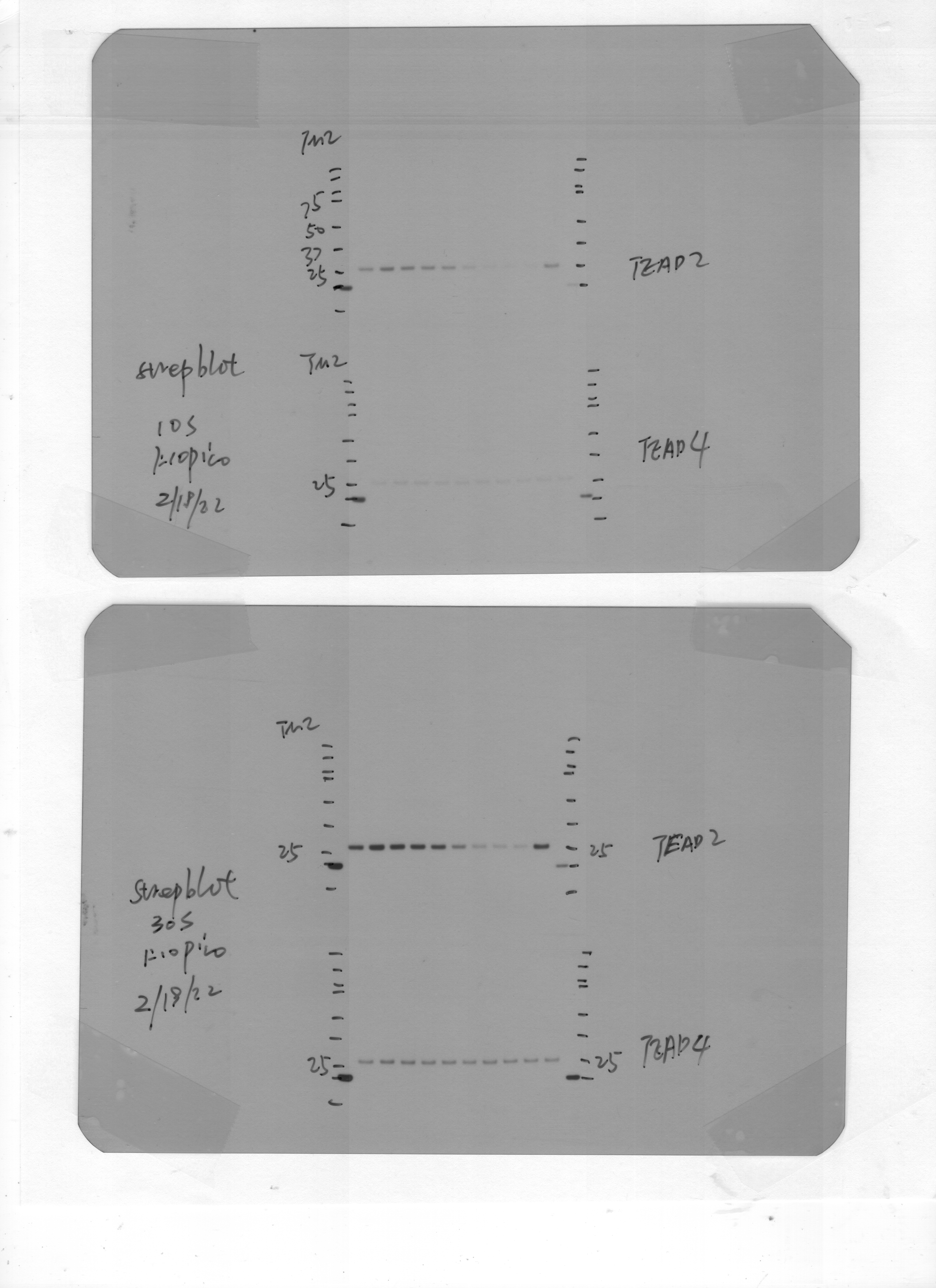

Supplement: Figure 1—source data 1. [file elife-80210-fig1-data1.zip › Figure 1C/TEAD2-IC50-strep-2.tif]

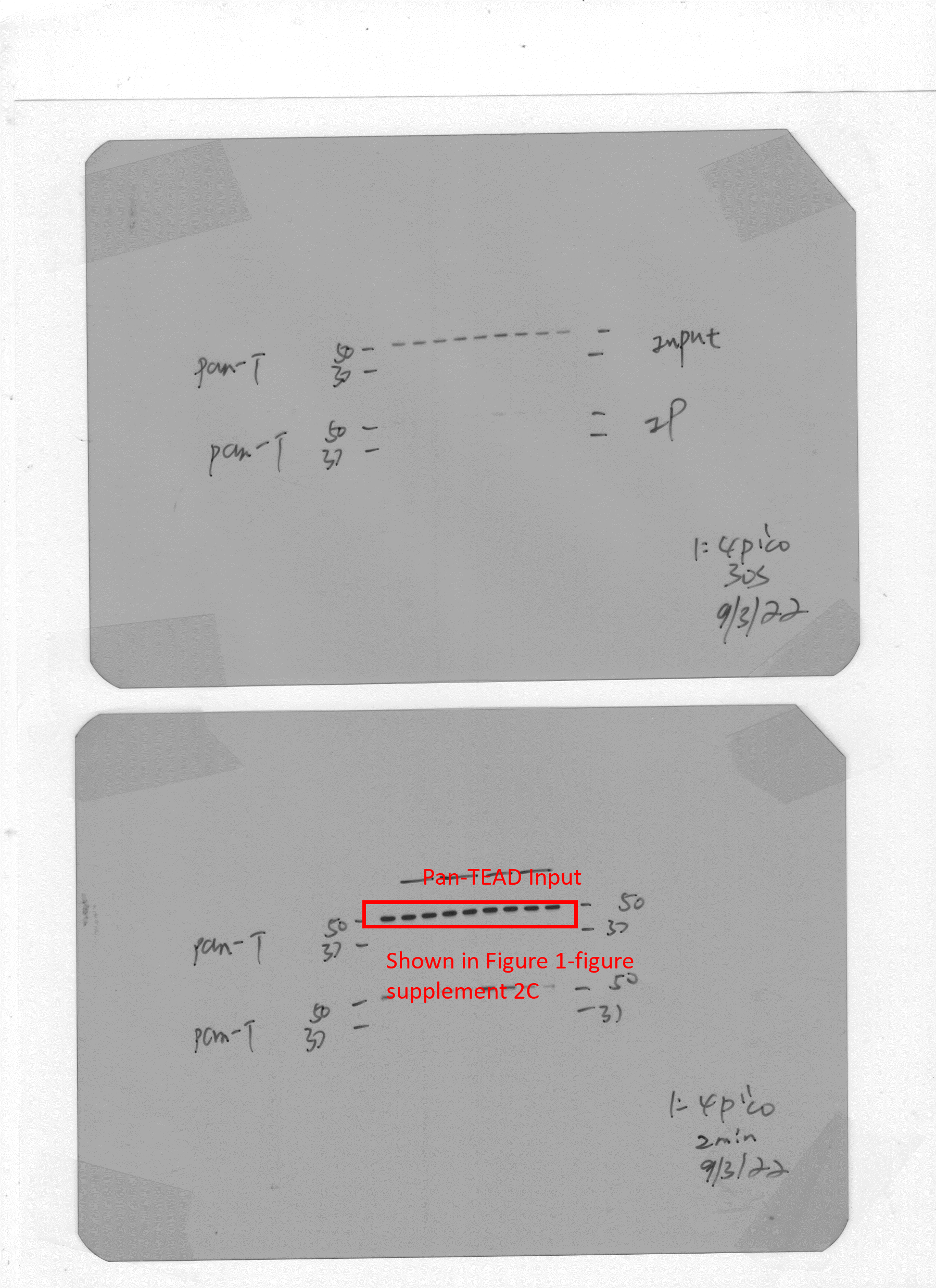

Supplement: Figure 1—figure supplement 2—source data 1. [file elife-80210-fig1-figsupp2-data1.zip › Figure 1-figure suppment 2D/pan-TEAD Input-labeled.tif]

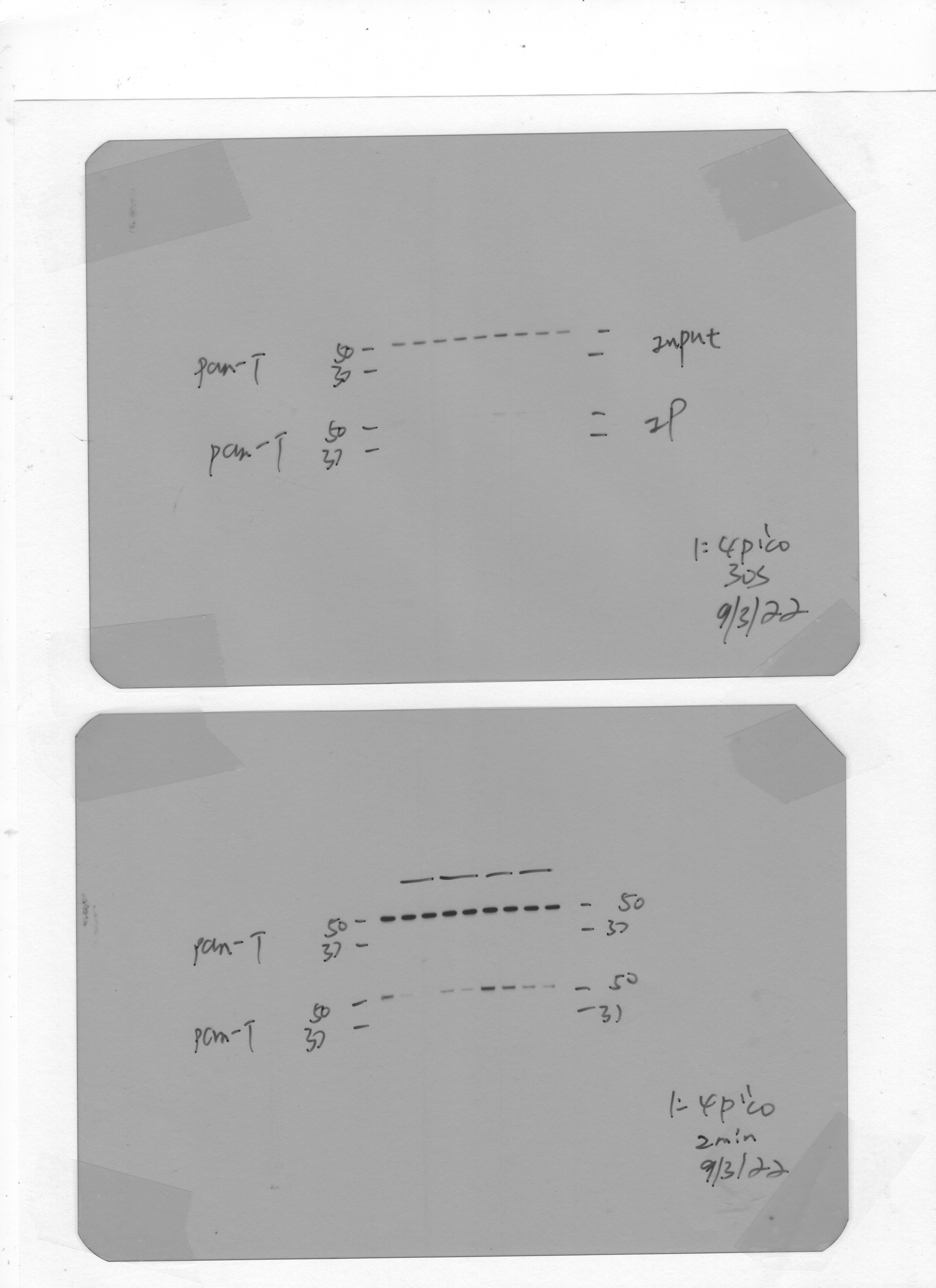

Supplement: Figure 1—figure supplement 2—source data 1. [file elife-80210-fig1-figsupp2-data1.zip › Figure 1-figure suppment 2D/pan-TEAD Input.tif]

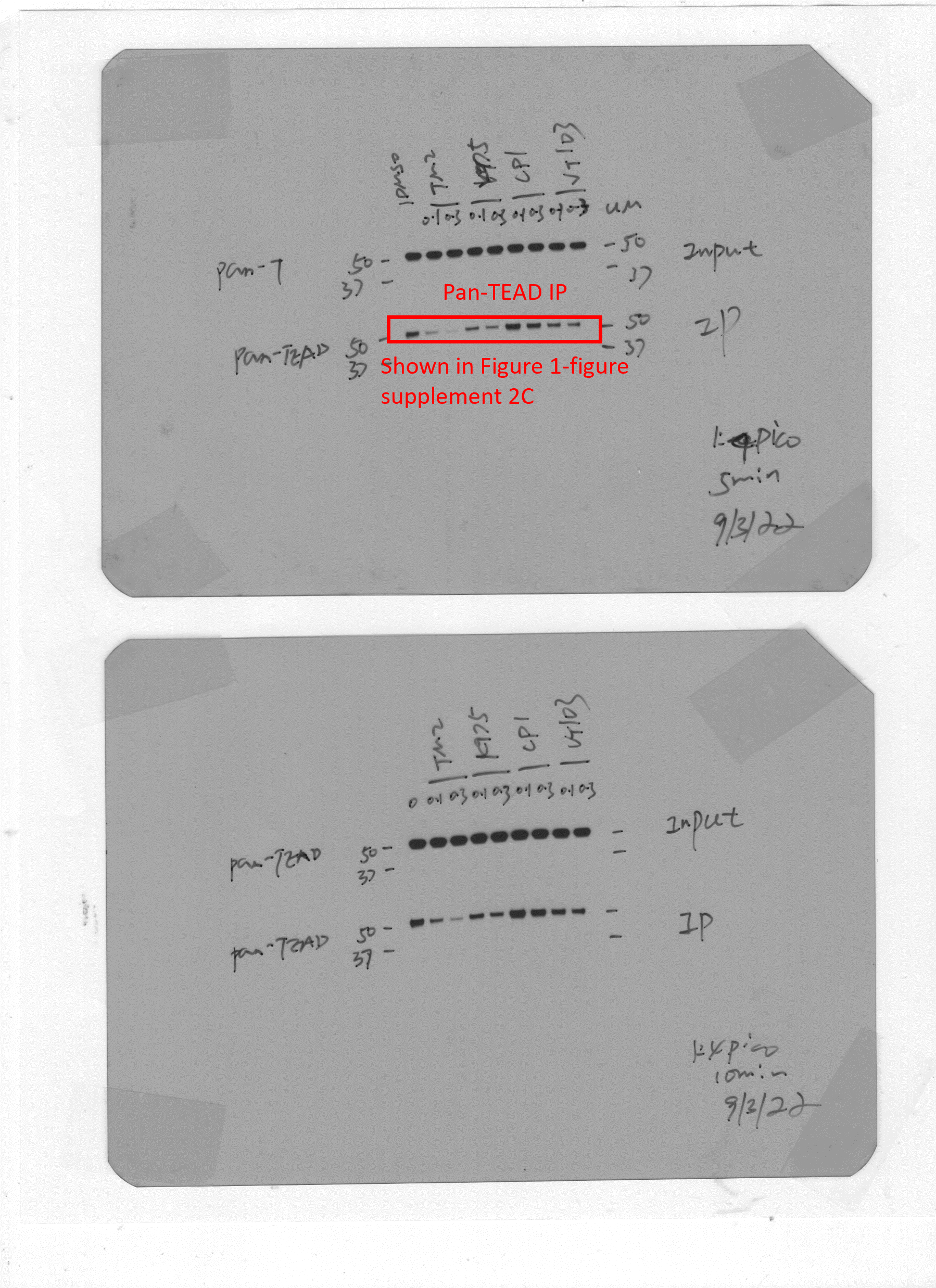

Supplement: Figure 1—figure supplement 2—source data 1. [file elife-80210-fig1-figsupp2-data1.zip › Figure 1-figure suppment 2D/Pan-TEAD IP-labeled.tif]

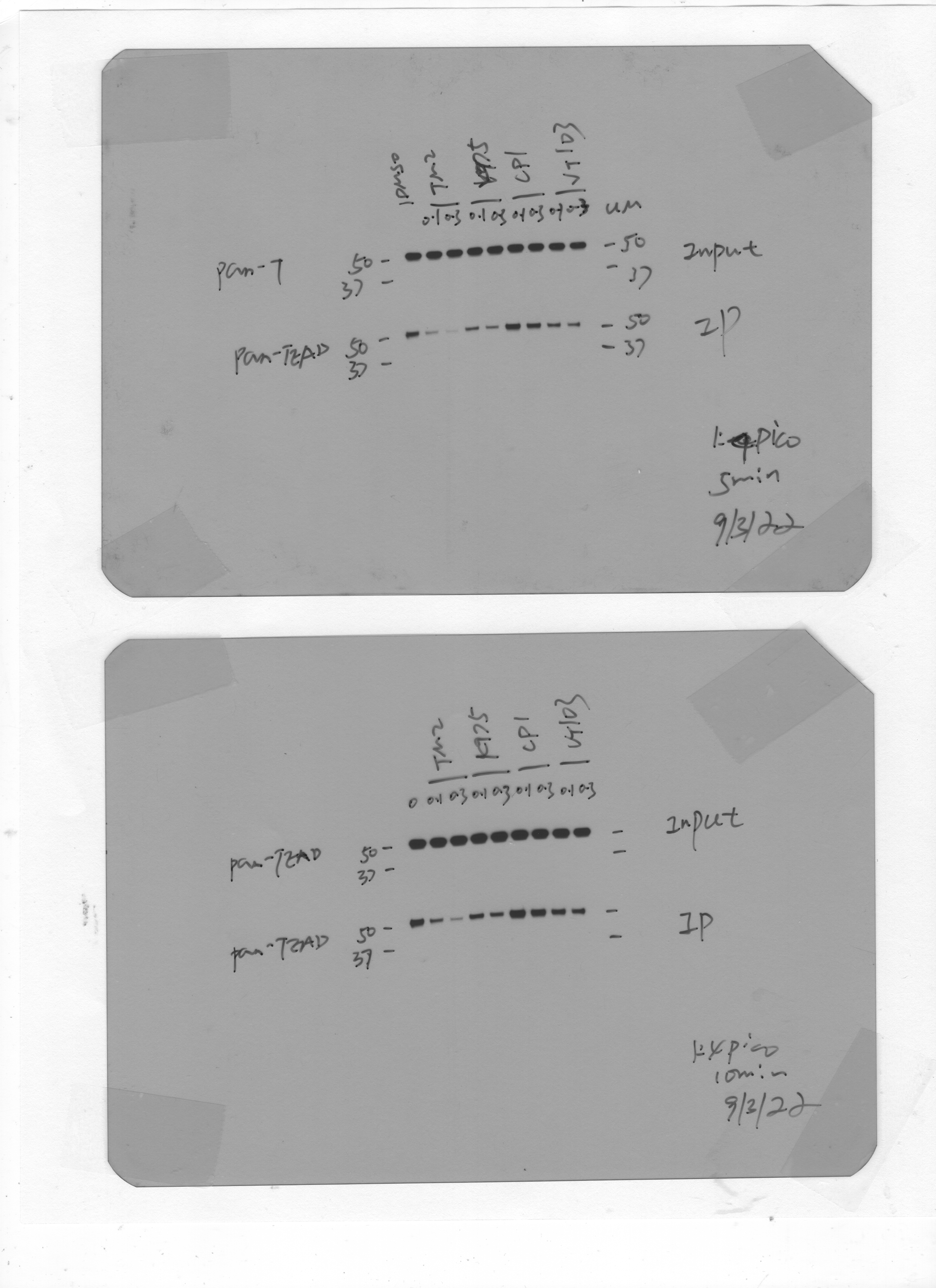

Supplement: Figure 1—figure supplement 2—source data 1. [file elife-80210-fig1-figsupp2-data1.zip › Figure 1-figure suppment 2D/Pan-TEAD IP.tif]

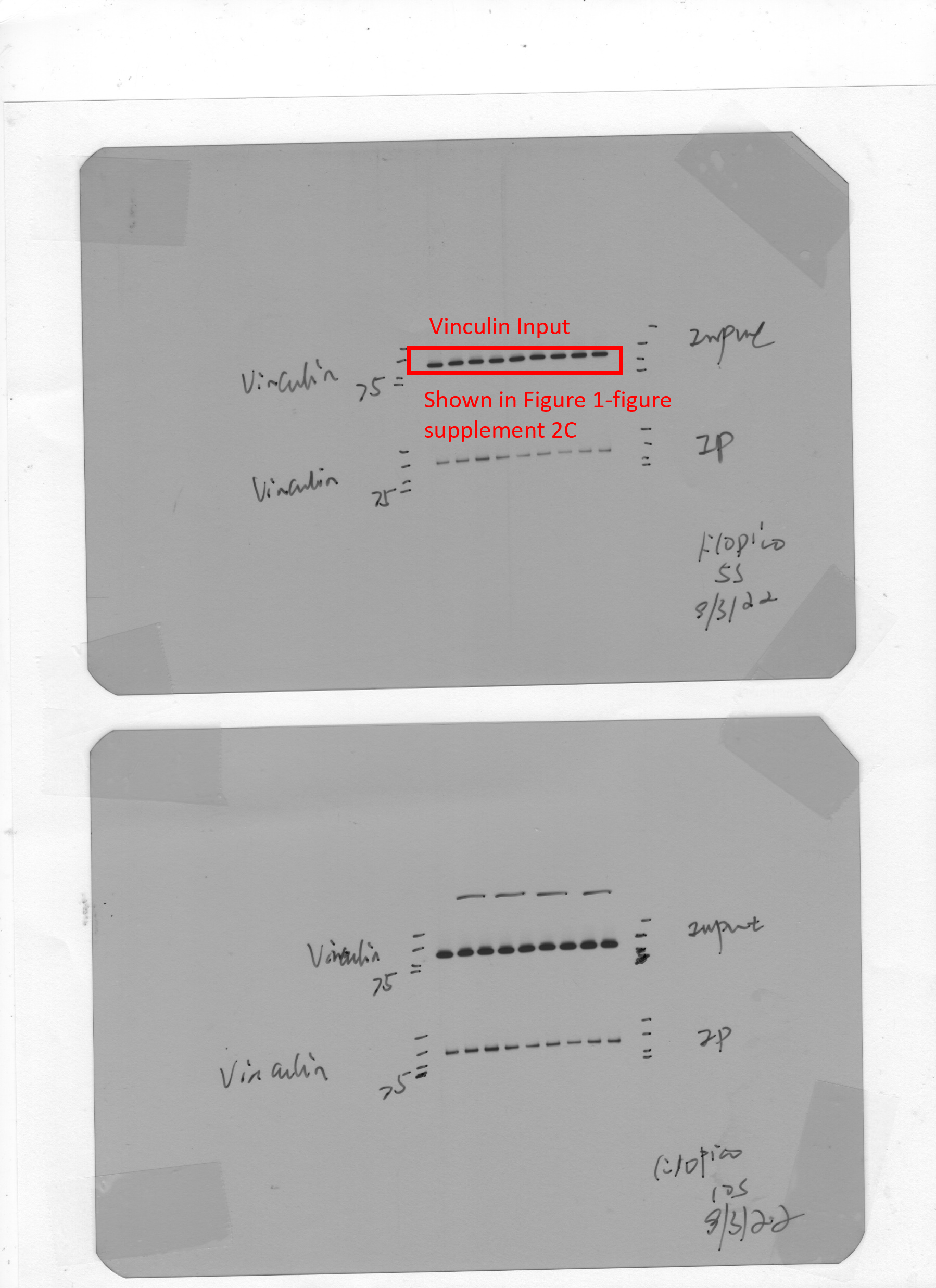

Supplement: Figure 1—figure supplement 2—source data 1. [file elife-80210-fig1-figsupp2-data1.zip › Figure 1-figure suppment 2D/Vinculin Input-labeled.tif]

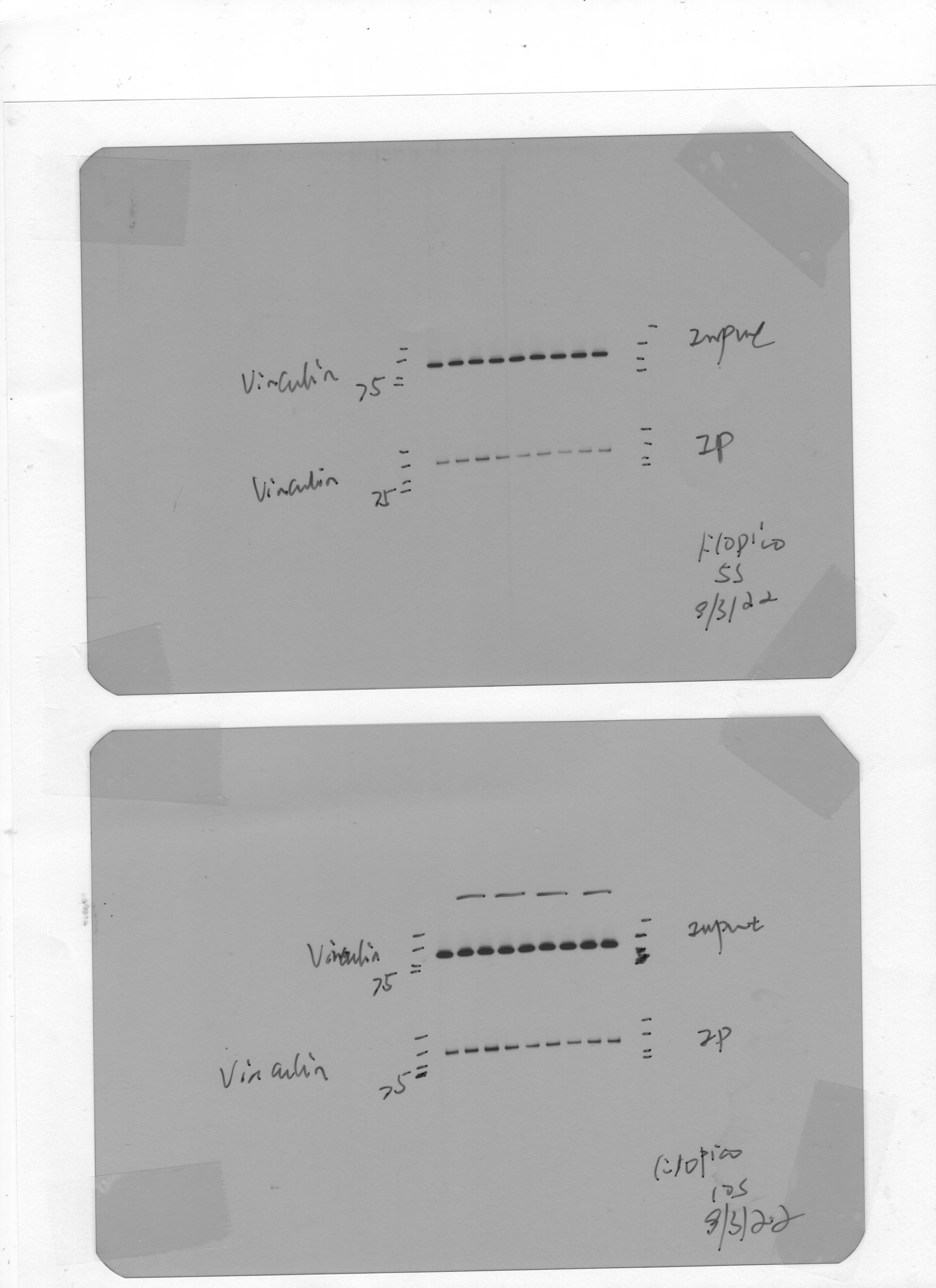

Supplement: Figure 1—figure supplement 2—source data 1. [file elife-80210-fig1-figsupp2-data1.zip › Figure 1-figure suppment 2D/Vinculin Input.tif]

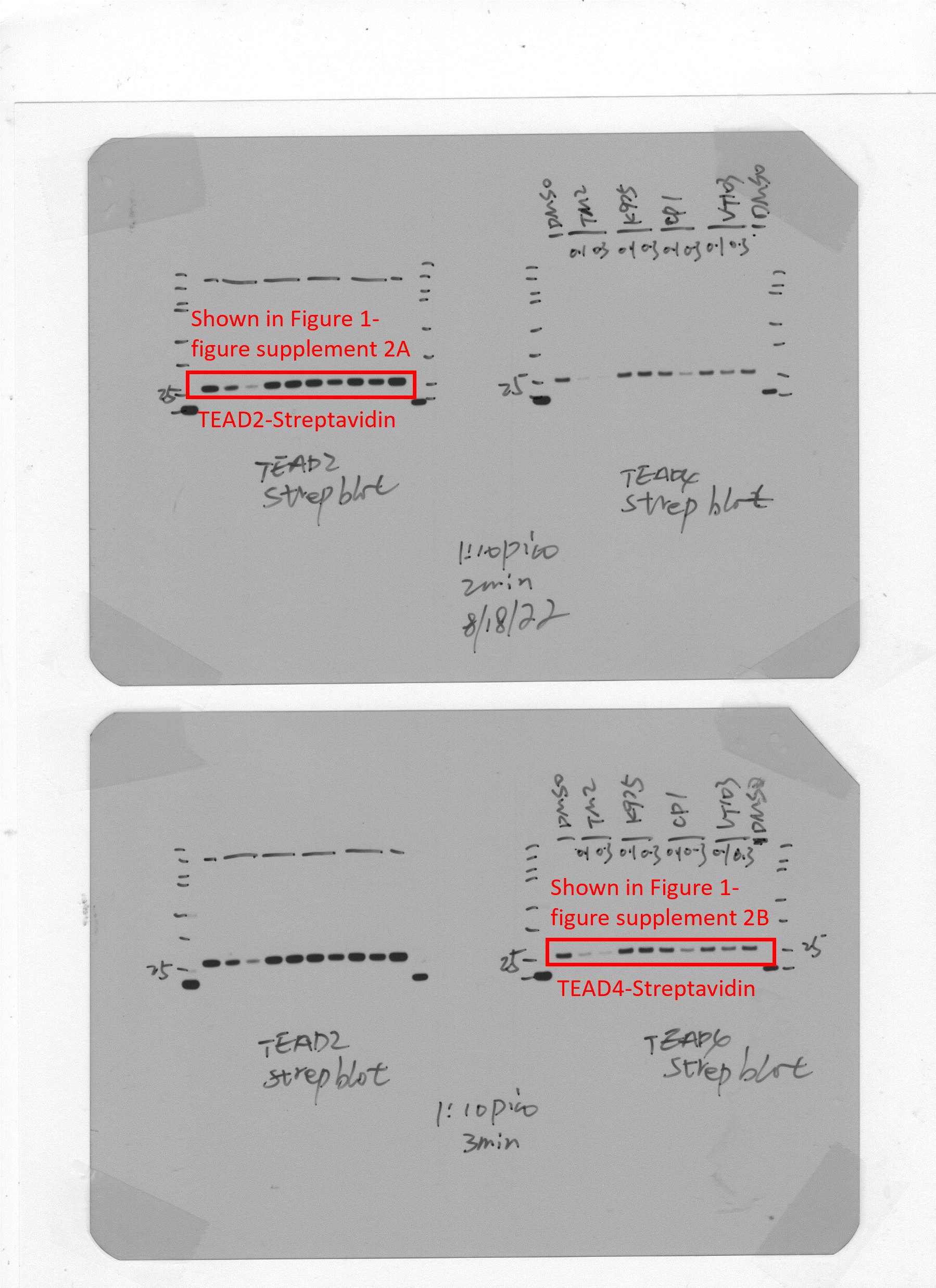

Supplement: Figure 1—figure supplement 2—source data 1. [file elife-80210-fig1-figsupp2-data1.zip › Figure 1-figure suppment 2A and B/TEAD2 and TEAD4 strep - labled.tif]

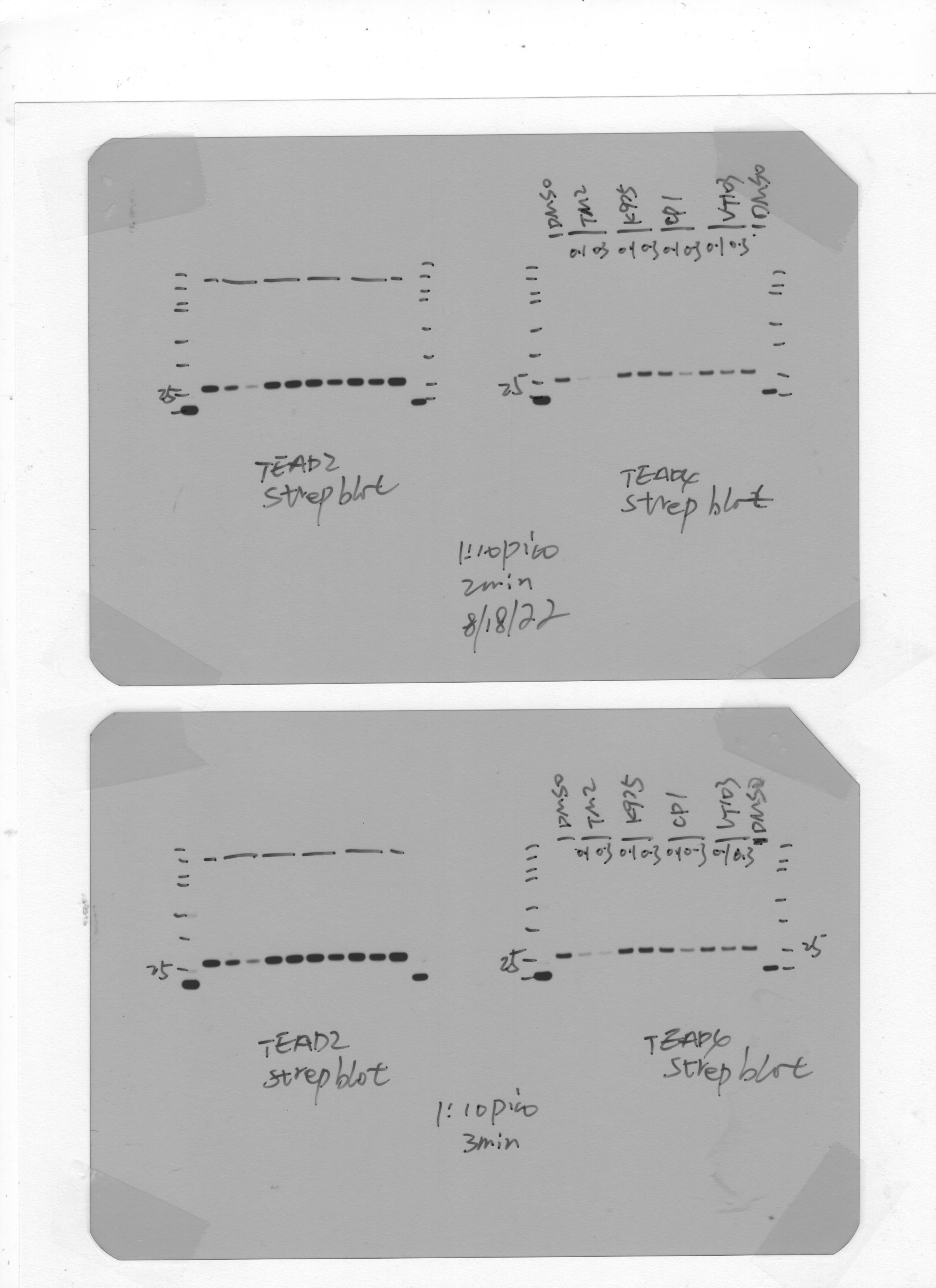

Supplement: Figure 1—figure supplement 2—source data 1. [file elife-80210-fig1-figsupp2-data1.zip › Figure 1-figure suppment 2A and B/TEAD2 and TEAD4 strep.tif]

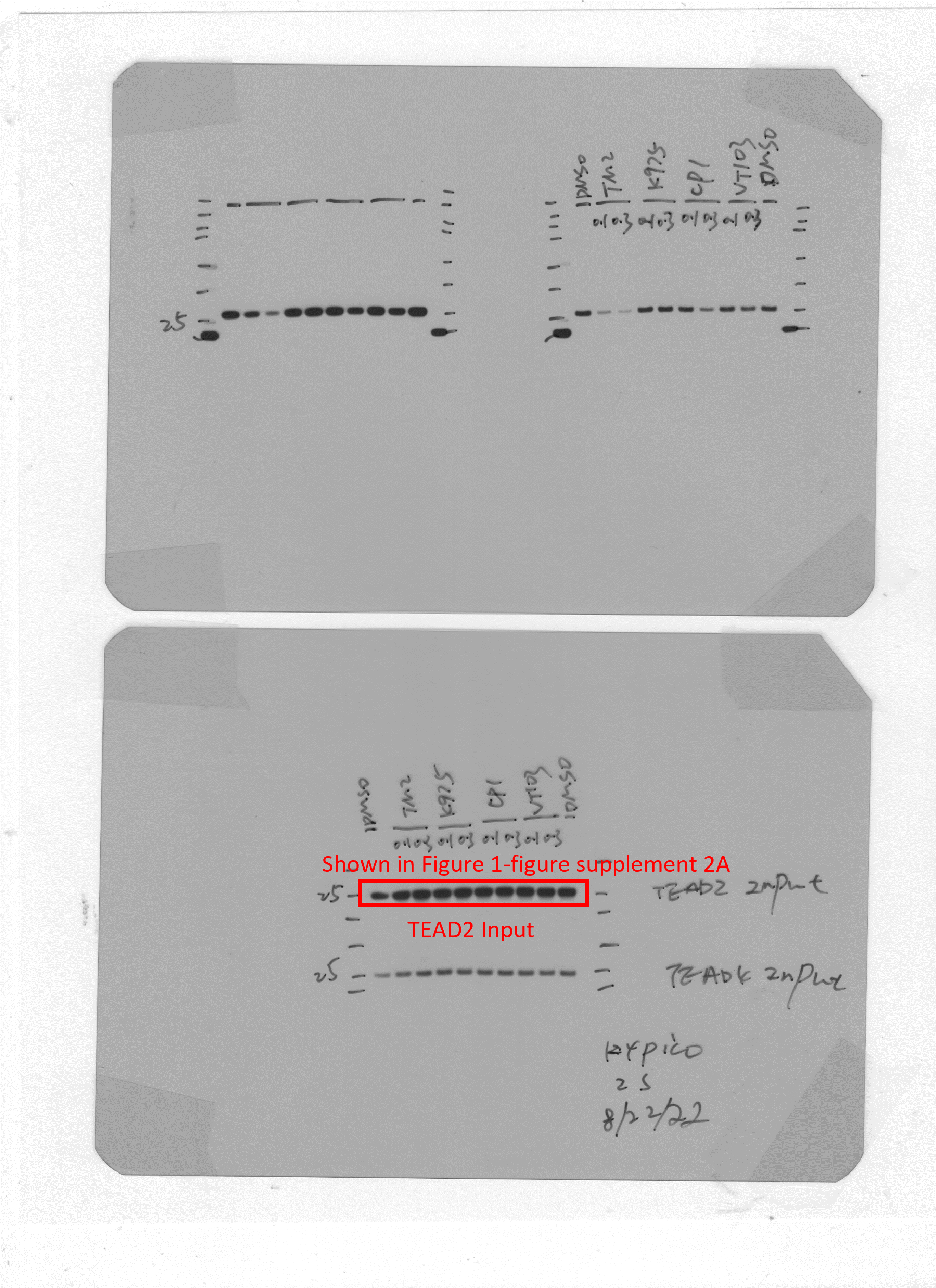

Supplement: Figure 1—figure supplement 2—source data 1. [file elife-80210-fig1-figsupp2-data1.zip › Figure 1-figure suppment 2A and B/TEAD2 Input-labeled.tif]

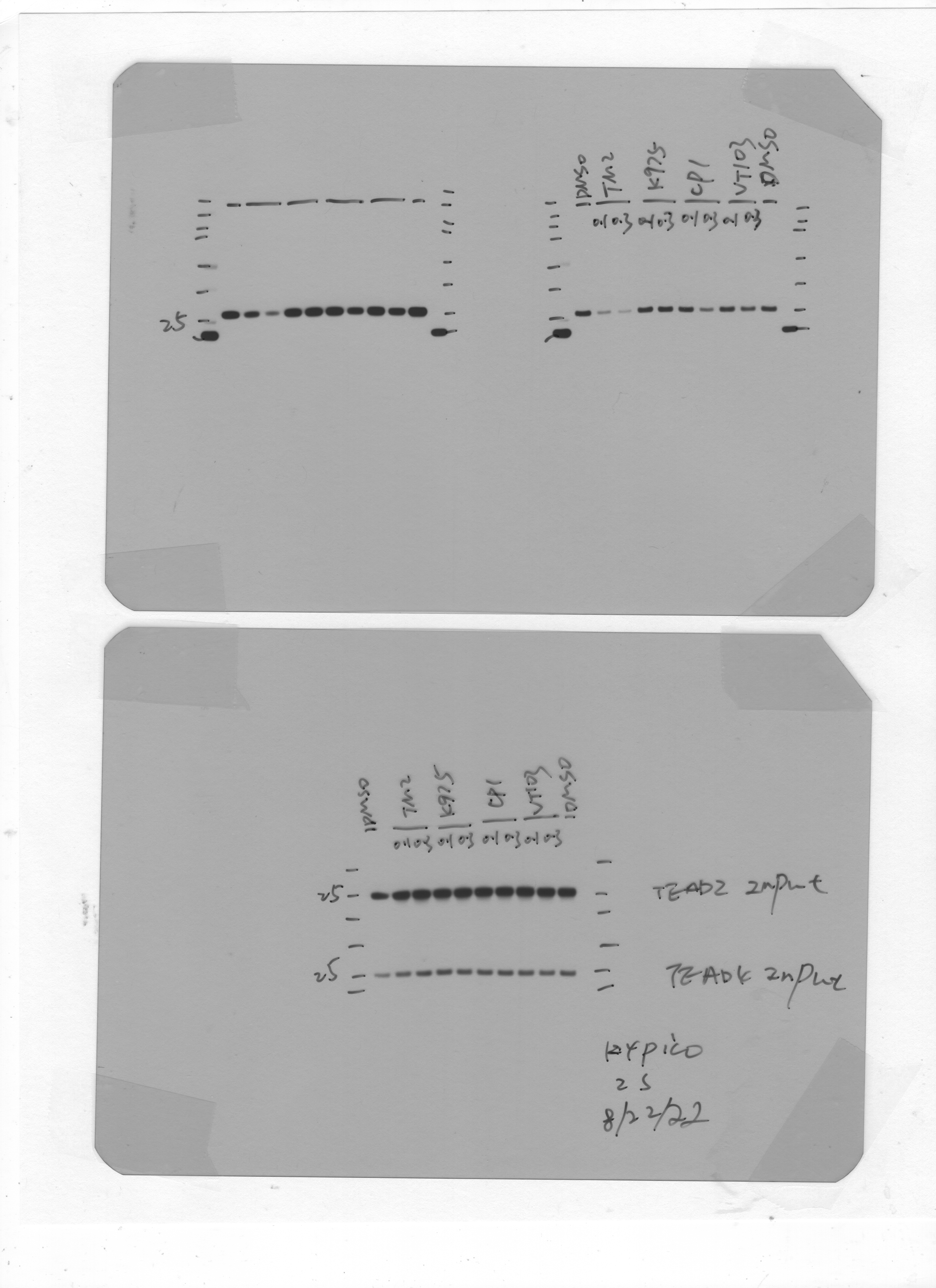

Supplement: Figure 1—figure supplement 2—source data 1. [file elife-80210-fig1-figsupp2-data1.zip › Figure 1-figure suppment 2A and B/TEAD2 Input.tif]

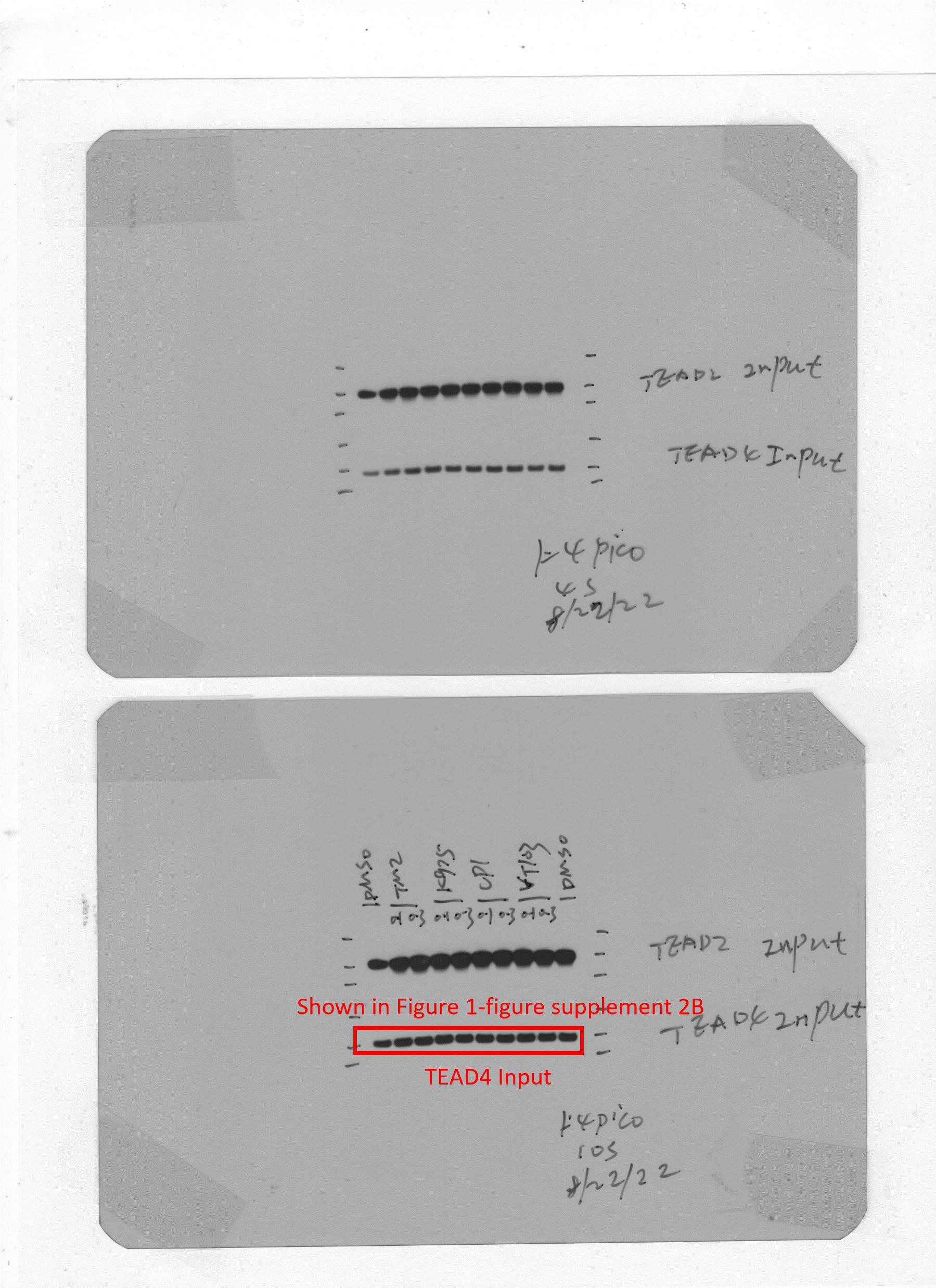

Supplement: Figure 1—figure supplement 2—source data 1. [file elife-80210-fig1-figsupp2-data1.zip › Figure 1-figure suppment 2A and B/TEAD4 Input-labeled.tif]

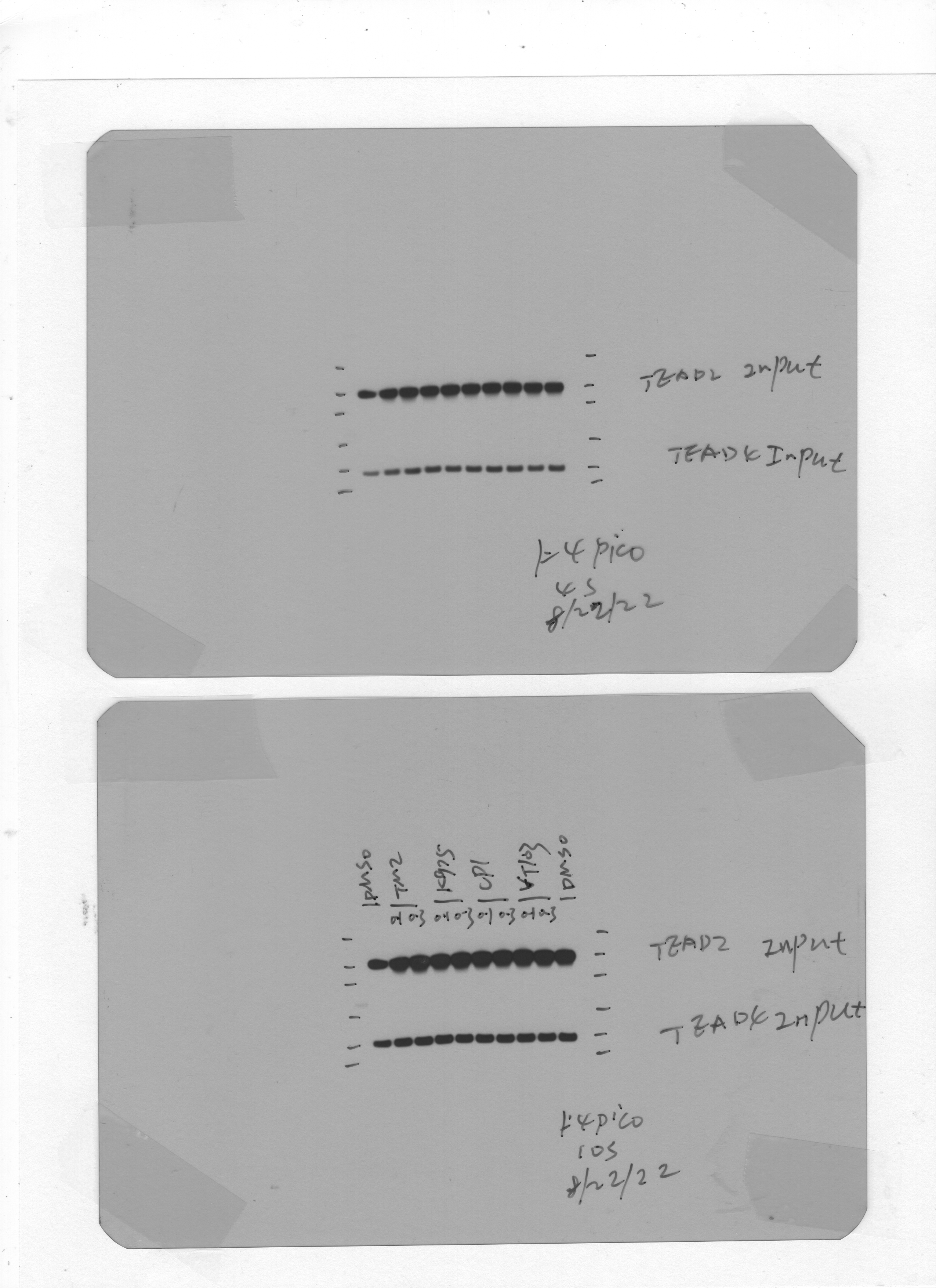

Supplement: Figure 1—figure supplement 2—source data 1. [file elife-80210-fig1-figsupp2-data1.zip › Figure 1-figure suppment 2A and B/TEAD4 Input.tif]

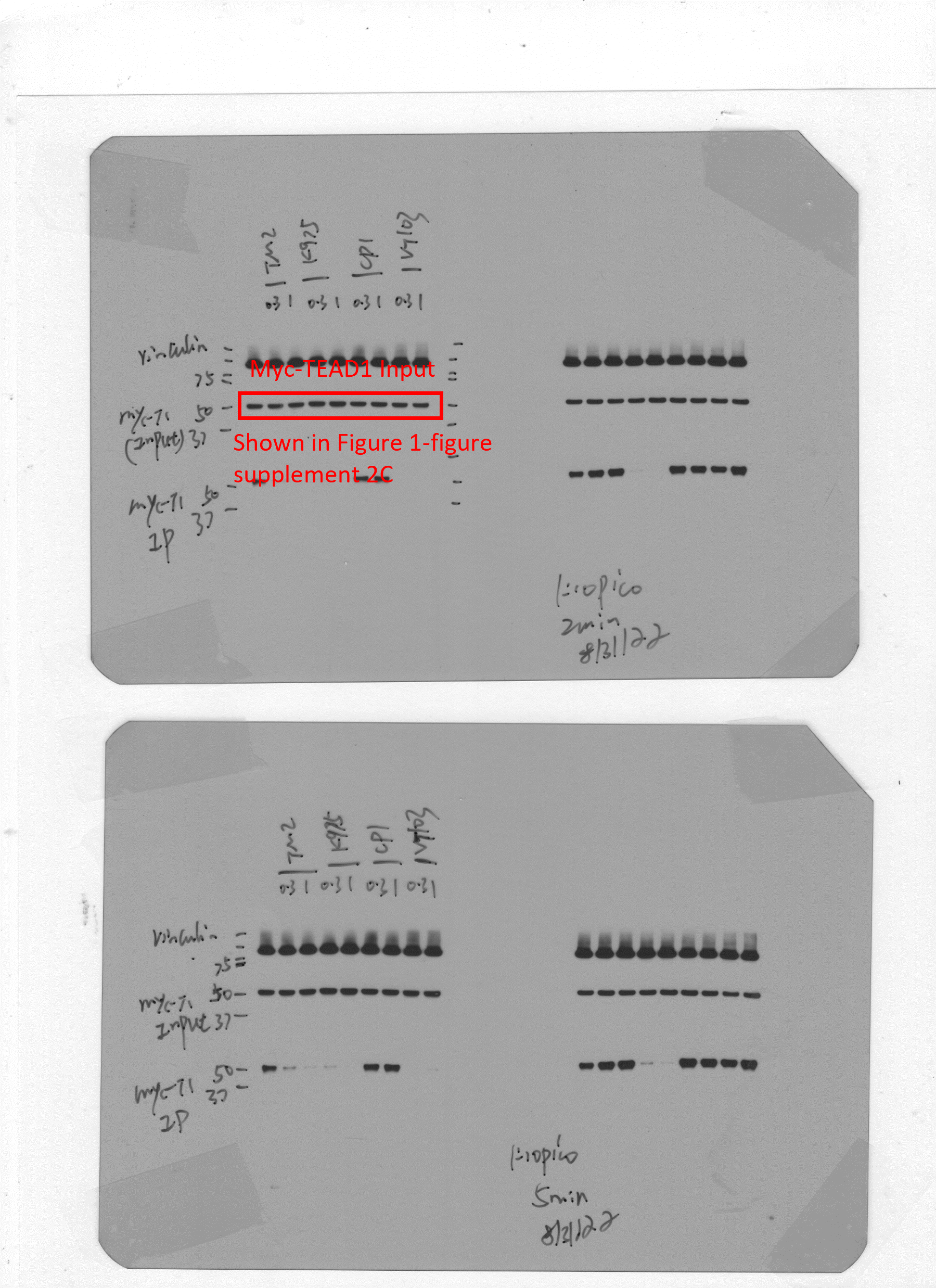

Supplement: Figure 1—figure supplement 2—source data 1. [file elife-80210-fig1-figsupp2-data1.zip › Figure 1-figure suppment 2C/Myc-TEAD1 Input-labeled.tif]

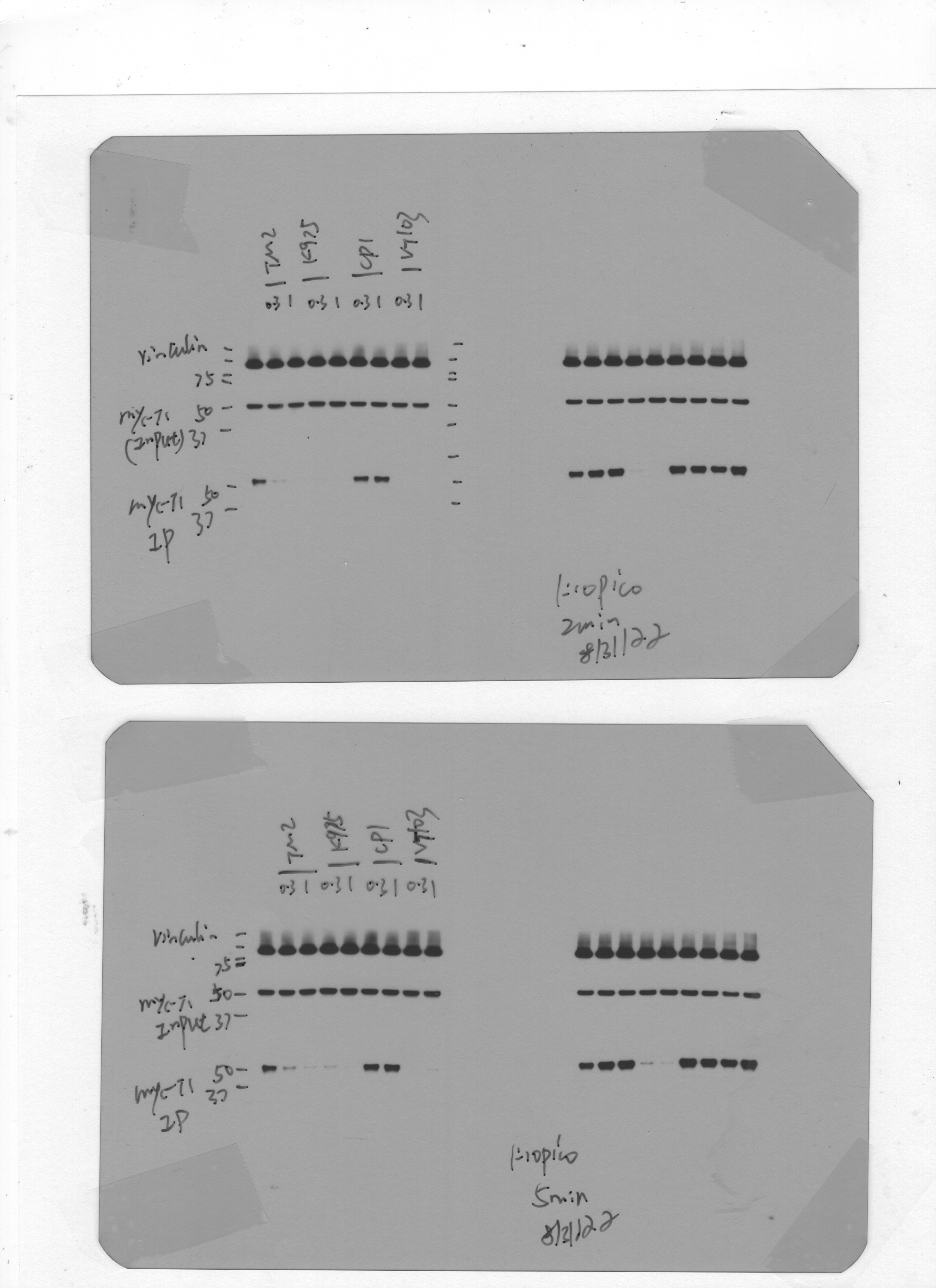

Supplement: Figure 1—figure supplement 2—source data 1. [file elife-80210-fig1-figsupp2-data1.zip › Figure 1-figure suppment 2C/Myc-TEAD1 Input.tif]

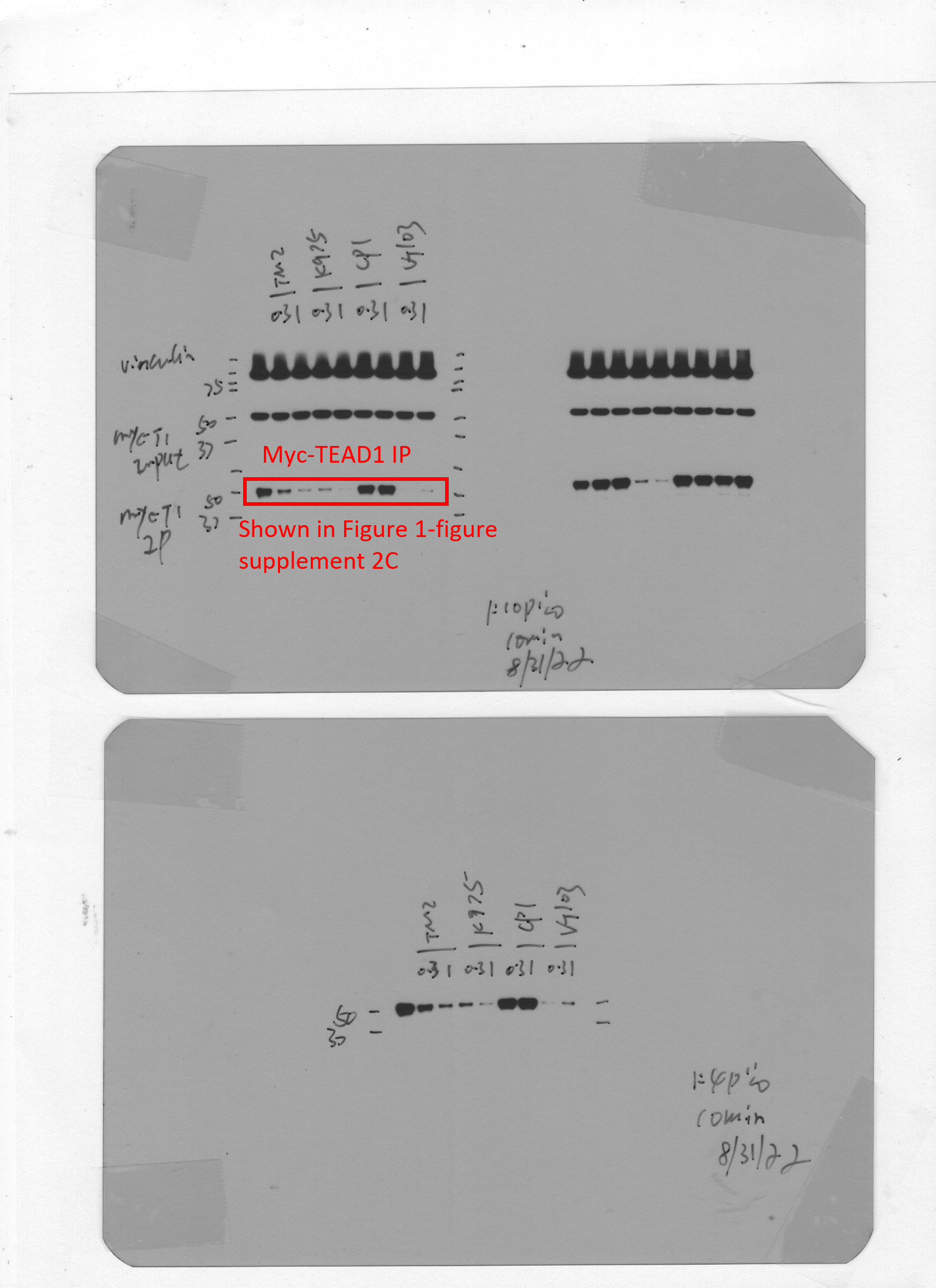

Supplement: Figure 1—figure supplement 2—source data 1. [file elife-80210-fig1-figsupp2-data1.zip › Figure 1-figure suppment 2C/Myc-TEAD1 IP-labeled.tif]

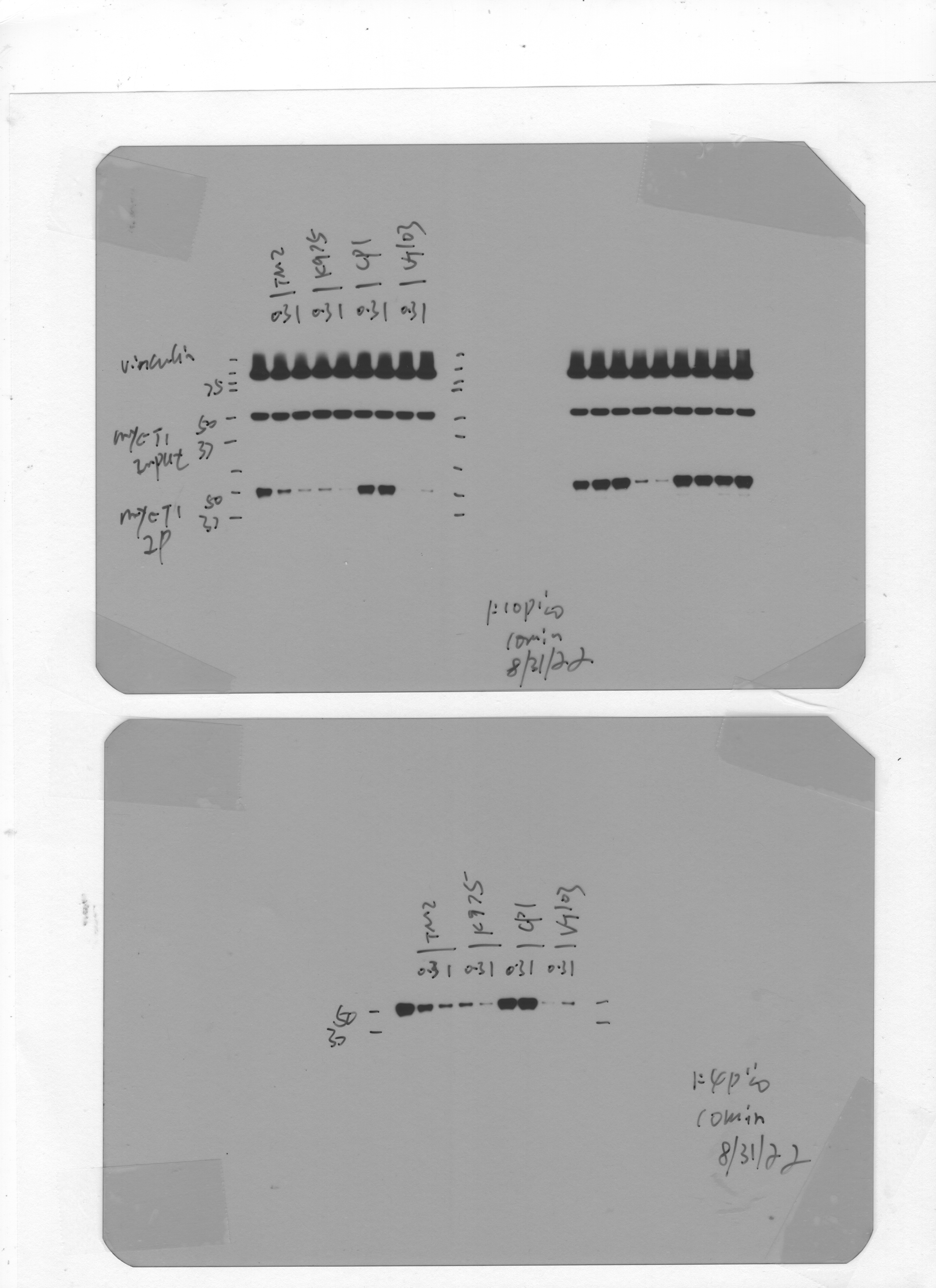

Supplement: Figure 1—figure supplement 2—source data 1. [file elife-80210-fig1-figsupp2-data1.zip › Figure 1-figure suppment 2C/Myc-TEAD1 IP.tif]

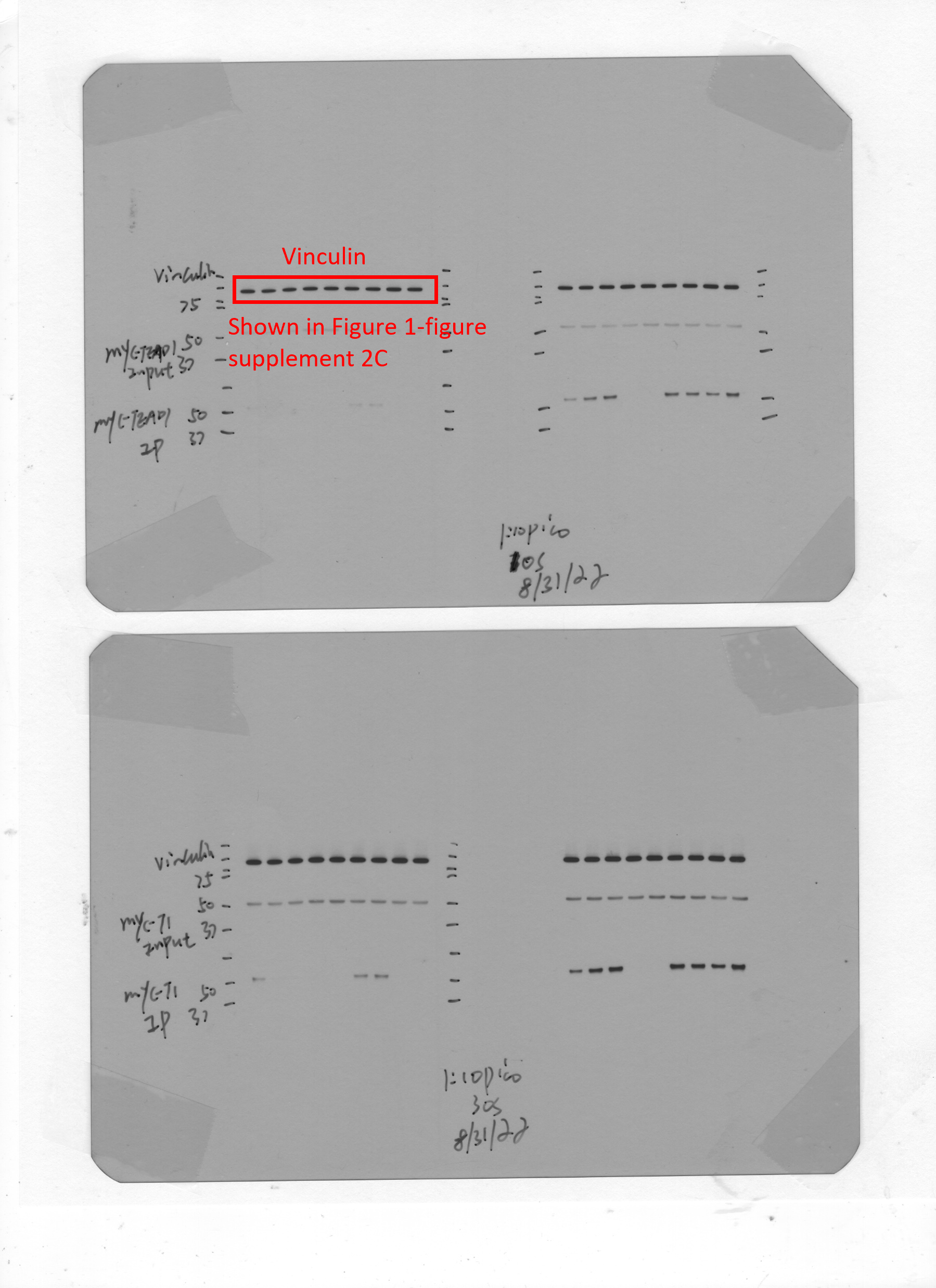

Supplement: Figure 1—figure supplement 2—source data 1. [file elife-80210-fig1-figsupp2-data1.zip › Figure 1-figure suppment 2C/Vinculin-labeled.tif]

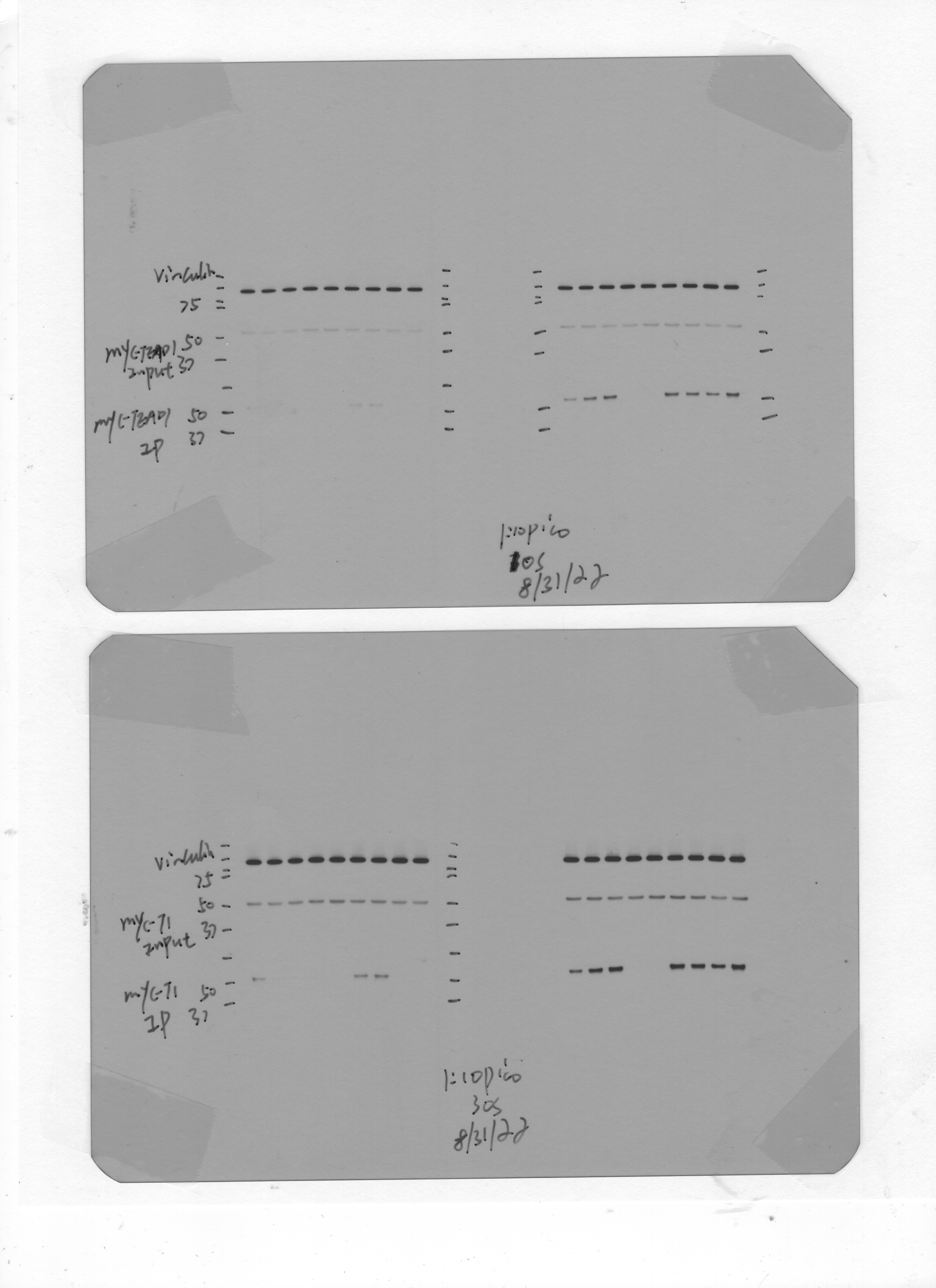

Supplement: Figure 1—figure supplement 2—source data 1. [file elife-80210-fig1-figsupp2-data1.zip › Figure 1-figure suppment 2C/Vinculin.tif]

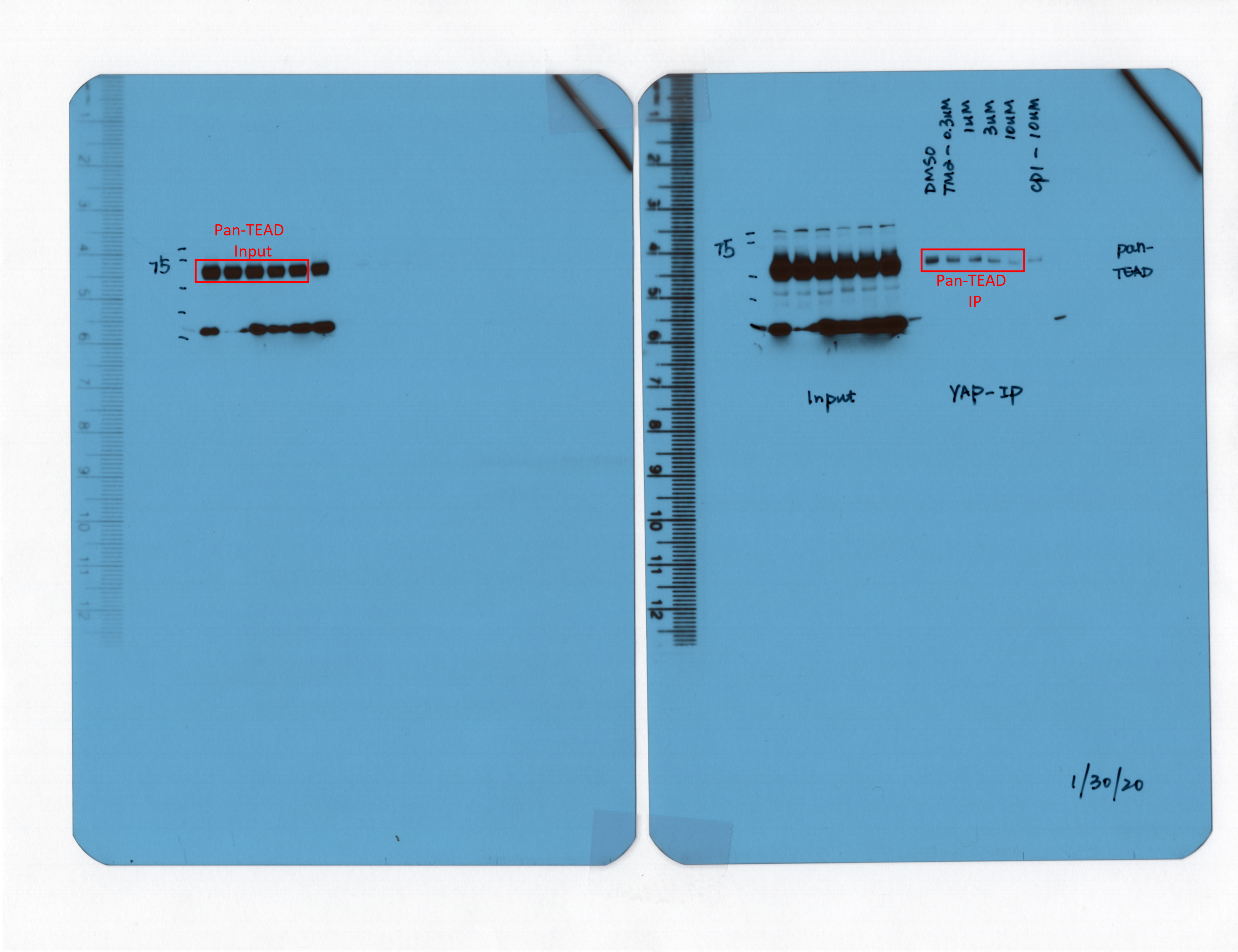

Supplement: Figure 3—source data 1. [file elife-80210-fig3-data1.zip › Figure 3A/1-labeled.tif]

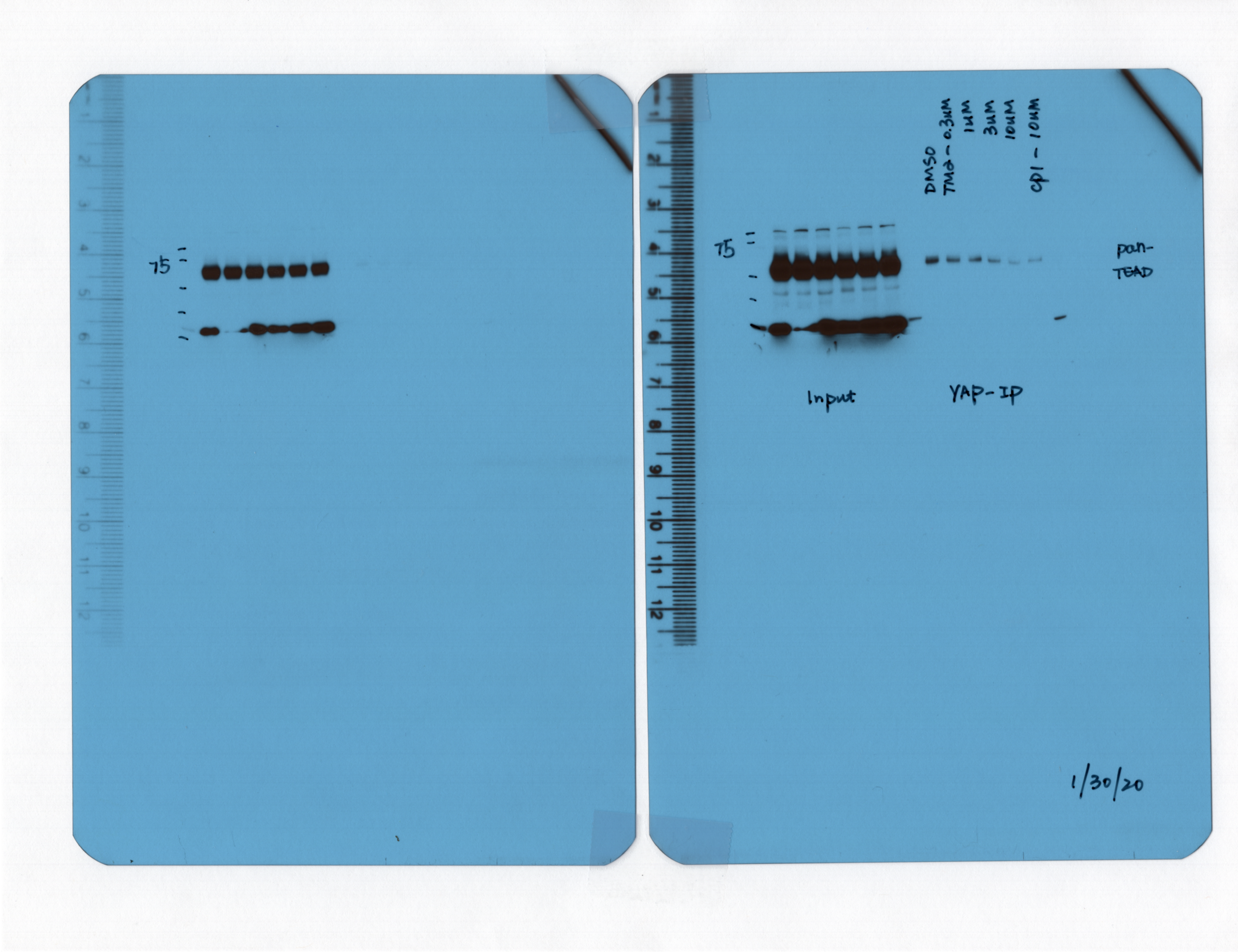

Supplement: Figure 3—source data 1. [file elife-80210-fig3-data1.zip › Figure 3A/1.tif]

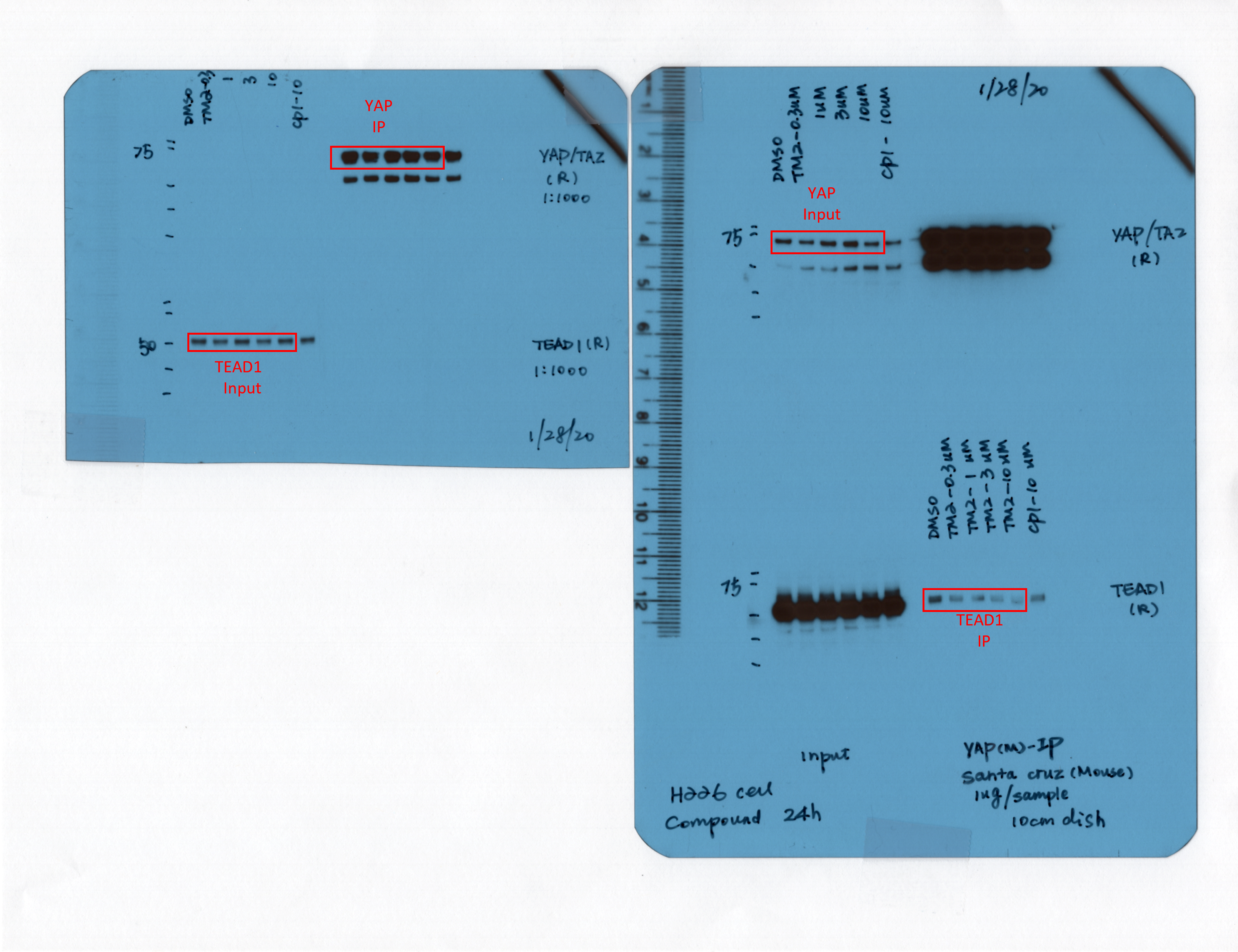

Supplement: Figure 3—source data 1. [file elife-80210-fig3-data1.zip › Figure 3A/2-labeled.tif]
