# Supplementary material for: Discovery of a new class of reversible TEA domain transcription factor inhibitors with a novel binding mode
Source: eLife. 2022 Nov 18;11:e80210. doi: 10.7554/eLife.80210 (PMC9728997; doi:10.7554/eLife.80210)
Supplement: Figure 2—source data 1. [file elife-80210-fig2-data1.zip › Figure 2-Source data 1.docx]

|  | **TEAD2-TM2** |
| --- | --- |
| **Data collection** |  |
| Wavelength (Å) | 0.979 |
| Resolution (Å^2^) | 50.00-2.40 (2.44-2.40) |
| Space group | *C*2 |
| Unit cell dimensions |  |
| *a*, *b*, *c* (Å) | 124.07, 62.29, 79.91 |
| *α*, *β*, *γ* (°) | 90.0, 117.7, 90.0 |
| Redundancy | 3.6 (2.8) |
| Completeness (%) | 97.0 (83.7) |
| Reflections (unique) | 20, 774 |
| *I*/*σ_I_* | 24.1 (1.5) |
| *R*_sym_ (%) | 5.2 (68.0) |
| *R*_pim_ (%) | 3.1 (45.6) |
| CC_1/2_^a^ | 0.720 |
| **Refinement** |  |
| No. of non-hydrogen atoms | 3, 393 |
| Protein | 3, 299 |
| Ligand | 64 |
| Water | 30 |
| Average *B* factor (Å^2^) | 48.9 |
| Protein | 49.0 |
| Ligand | 47.2 |
| Water | 42.7 |
| *R*work/*R*free (%) | 18.38/23.46 |
| RMSDs |  |
| Bond length (Å) | 0.008 |
| Bond angle (°) | 1.099 |
| Favored/allowed/outliers (%) | 92.75/7.25/0.00 |

Values for the highest resolution shell are given in parentheses.

^a^CC_1/2_ values shown are for the highest resolution shell.
